# Discovery of Natural Products as Novel and Potent FXR Antagonists by Virtual Screening

**DOI:** 10.3389/fchem.2018.00140

**Published:** 2018-04-30

**Authors:** Yanyan Diao, Jing Jiang, Shoude Zhang, Shiliang Li, Lei Shan, Jin Huang, Weidong Zhang, Honglin Li

**Affiliations:** ^1^Shanghai Key Laboratory of New Drug Design, State Key Laboratory of Bioreactor Engineering, School of Pharmacy, East China University of Science and Technology, Shanghai, China; ^2^Department of Phytochemistry, School of Pharmacy, Second Military Medical University, Shanghai, China

**Keywords:** FXR, antagonist, virtual screening, molecular docking, similarity searching, natural product

## Abstract

Farnesoid X receptor (FXR) is a member of nuclear receptor family involved in multiple physiological processes through regulating specific target genes. The critical role of FXR as a transcriptional regulator makes it a promising target for diverse diseases, especially those related to metabolic disorders such as diabetes and cholestasis. However, the underlying activation mechanism of FXR is still a blur owing to the absence of proper FXR modulators. To identify potential FXR modulators, an in-house natural product database (NPD) containing over 4,000 compounds was screened by structure-based virtual screening strategy and subsequent hit-based similarity searching method. After the yeast two-hybrid (Y2H) assay, six natural products were identified as FXR antagonists which blocked the CDCA-induced SRC-1 association. The IC_50_ values of compounds **2a**, a diterpene bearing polycyclic skeleton, and **3a**, named daphneone with chain scaffold, are as low as 1.29 and 1.79 μM, respectively. Compared to the control compound guggulsterone (IC_50_ = 6.47 μM), compounds **2a** and **3a** displayed 5- and 3-fold higher antagonistic activities against FXR, respectively. Remarkably, the two representative compounds shared low topological similarities with other reported FXR antagonists. According to the putative binding poses, the molecular basis of these antagonists against FXR was also elucidated in this report.

## Introduction

The farnesoid X receptor (FXR, NR1H4), a member of the metabolic nuclear receptor superfamily, regulates the expressions and activities of a broad spectrum of genes. Since 1995 when FXR was isolated from a rat cDNA library for the first time (Forman et al., 1995), the studies on the physiological functions of FXR have been appealing and challenging. FXR is conserved from teleost fish to human beings (Maglich et al., 2003) and is abundantly expressed in liver, intestine, and kidney. As the endogenous receptor of bile acids, FXR can be activated by chenodeoxycholic acid (CDCA), lithocholic acid (LCA), deoxycholic acid (DCA), and many other bile acids (Makishima et al., 1999). In addition, FXR is also reported to exert regulatory roles in lipoprotein and glucose homeostasis, fatty acid and triglyceride synthesis, liver regeneration, and bacterial growth in the intestine (Lee et al., 2006; Wang et al., 2008). All these accumulating data make FXR a promising pharmaceutical target for multiple diseases, especially those related to metabolic disorders such as diabetes and cholestasis (Schaap et al., 2014; Gonzalez et al., 2016; Yuan and Li, 2016; Filho et al., 2017).

As a typical nuclear receptor, FXR shares common structural characteristics with other members of this superfamily, which comprises a highly conserved DNA-binding domain (DBD), a moderately conserved ligand-binding domain (LBD), and a ligand-dependent transcriptional activation domain (AF-2) (Pellicciari et al., 2005). Upon the binding of proper ligand to the LBD, FXR will undergo a conformational change, which is critical to determine whether a coactivator or a corepressor binds efficiently to the AF-2 motif. If activated by appropriate agonists, the recruitment of coactivators (such as SRC-1, DRIP, and PRMT) to FXR occurs, which further up- or down-regulates the expressions of certain target genes. While for antagonists, the association of FXR with activators will be hindered (Lew et al., 2004). Although it is widely accepted that FXR participates in many biological processes, owing to the diversity and complexity of target genes involved in the FXR signaling pathways (Zhang and Edwards, 2008), the physiological functions of FXR haven't been clearly defined. Therefore, it is still an essential step to identify potential FXR modulators, which may contribute to the elucidation of physiological effects of FXR and provide novel opportunities for the treatment of metabolic diseases by targeting FXR.

**Figure 1:**
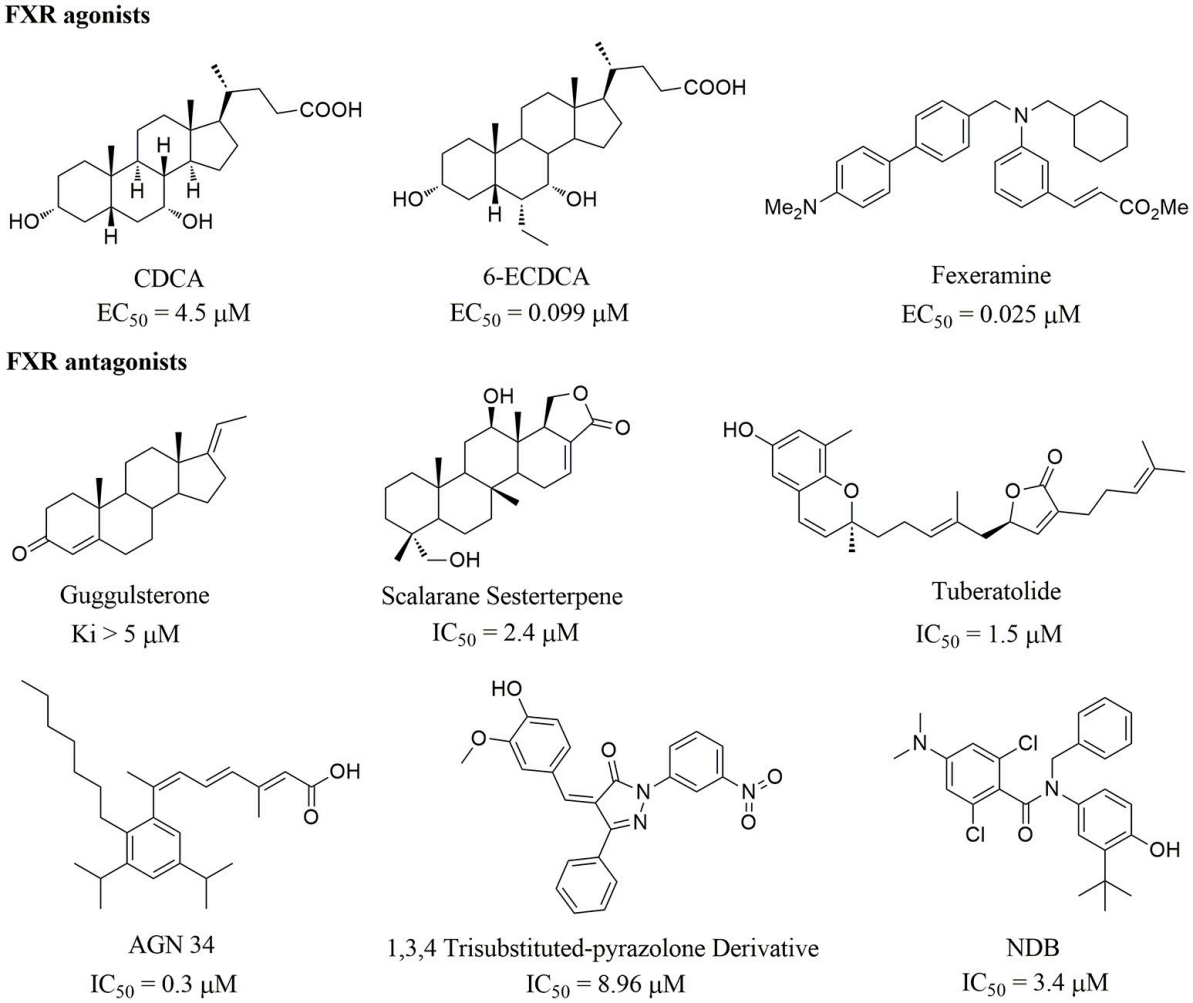
Selected structures of the reported FXR modulators.

Apart from the natural bile acid ligands with steroidal skeleton, over 700 structurally diverse FXR modulators have also been identified (Gaulton et al., 2017), most of which function as agonists (Carotti et al., 2014). Obeticholic acid (6α-ethyl-chenodeoxycholic acid, 6-ECDCA), a semi-synthetic bile acid analog with highly potent FXR agonistic activity (EC_50_ = 0.099 μM) (Pellicciari et al., 2002), is the first FDA-approved drug that is used for treating primary biliary cholangitis (PBC) (Nevens et al., 2016). In contrast, the development of FXR antagonists, which are also useful chemical tools to unravel the physiological roles and relative clinical significance of FXR (Li et al., 2013), does not seem to be satisfactory due to the scanty number of potent FXR antagonists that have been reported so far. Guggulsterone, a natural product extracted from the resin of the guggul tree, is the most described FXR antagonist, with the ability of blocking the agonist-induced coactivator recruitment and decreasing the hepatic cholesterol in wild-type mice (Urizar et al., 2002). However, the researches on guggulsterone are still confined to preclinical and academic studies because of the complexity of its mechanism of action (Fiorucci et al., 2010; Yamada and Sugimoto, 2016). Although other natural or synthetic FXR antagonists have also been developed (Figure 1; Wu et al., 2002; Dussault et al., 2003; Nam et al., 2007; Choi et al., 2011; Huang et al., 2012; Xu et al., 2015), further pharmaceutically relevant activities were rarely reported. Herein, six natural products were identified as antagonists from an in-house natural product database (NPD) through virtual screening strategy and subsequent biological experiment validation. In the yeast two-hybrid (Y2H) assay (Fields and Sternglanz, 1994; Lin and Lai, 2017), these compounds could abolish CDCA-induced FXR activation at micromolar level. We hope the natural products revealed in this study will offer novel scaffolds for uncovering new FXR regulatory mechanism and provide insights into potential development for further discovery of FXR modulators.

## Materials and methods

### Structure-based virtual screening

#### Protein preparation

The crystal structures of FXR-LBD in complex with 6-ECDCA (Mi et al., 2003) (PDB code 1OSV, a dimer and chain B was used) and fexaramine (Downes et al., 2003) (PDB code 1OSH">1OSH, monomer) were obtained from the Protein Data Bank. The synthetically modified bile acid ligand 6-ECDCA was derived from the structure of CDCA, but showed almost 100-fold more potent FXR agonistic activity than CDCA and did not activate other nuclear receptors. Fexaramine is a synthetic non-steroidal FXR agonist, which was identified by optimization of a benzopyrane-based combinatorial derived library. The coactivators and all the water molecules were removed. Hydrogen atoms and charges were added during a brief relaxation performed using the “Protein Preparation Wizard” workflow in Maestro 10.1. After the hydrogen bond network was optimized, the crystal structure was minimized until the root-mean-square deviation (RMSD) between the minimized structure and the starting structure reached 0.3 Å with OPLS_2005 force field.

#### Glide docking

The grid-enclosing box was placed on the centroid of the crystallographic ligand in the optimized protein structure and defined to enclose residues located within 15.0 Å of the binding pocket. A scaling factor of 0.8 was set to van der Waals (VDW) radii of those receptor atoms with partial atomic charges of less than 0.15 to soften the nonpolar parts of the receptor. After addition of hydrogen atoms and ionization at a pH range of 5.0-9.0, the three-dimensional structures of compounds in the NPD were generated with Ligprep v3.3 module. Standard precision (SP) and extra precision (XP) approaches of Glide (Friesner et al., 2004; Halgren et al., 2004) were respectively adopted to dock the molecules into the binding site with the default parameters, and only the top one pose for each molecule were retained. After parallel Glide SP scorings using two different protein structures (PDB codes 1OSV and 1OSH), the top 500 docking poses were reserved for each docking calculation and subjected to XP calculation with a more precise scoring function, and the top 200 docking poses were retained, respectively, for further visual observation.

### Hit-based similarity searching

The similarity searching process was accomplished in Pipeline Pilot v7.5, and two of the most potent FXR antagonists **2a** and **3a** were used as query molecules, respectively. The Tanimoto coefficient (Tc) of similarity between the query molecule and the target molecule was calculated using SciTegic functional connectivity fingerprints of radius 4 (FCFP_4) (Bender et al., 2009). The minimum Tc was set to a low value of 0.3, to maximize the number of obtained analogs.

### SRC-1 recruitment assay

#### Materials

The restriction and modification enzymes in this work were obtained from New England Biolabs (Beijing, China). *P*-nitrophenyl α-D-galactopyranoside (PNP-α-Gal), yeast nitrogen base without amino acids, agar, PEG3350, dimethyl sulfoxide (DMSO), lithium acetate, and glucose were all purchased from Sigma (Shanghai, China). The yeast expression plasmids pGADT7 and pGBKT7 were from Clontech (Palo Alto, CA), and CDCA was from Merck. The dropout supplement free from leucine and tryptophan (-Leu/-Trp DO supplement) was bought from Takara, and Salmon Sperm DNA was obtained from invitrogen. The yeast strain AH109 was purchased from Clontech (Palo Alto, CA).

#### Plasmid construction

Based on the genome sequences of FXRα (GenBank accession no. NC 000012.10), human FXRα-LBD (200-473 AA) was sub-cloned into vector pGBKT-7 using NdeI and BamHI restrict enzyme sites. The primers used for PCR amplification were listed as follows: FXRα-LBD (sense) 5′-ATCATATG-GAAATTCAGTGTAAATCTAAGCG-3′, (anti-sense) 5′-ATGGATCCTCACTGCA-CGTCCCA-3′. The combination plasmid pGADT7-SRC-1 was prepared as described previously (Lin et al., 2008), by amplifying with the following primers: (sense) 5′-CAGAATTC- CATAACAATGACAGACTTTCA-3′ and (anti-sense) 5′-AAGGATCCCACCTTTA- CATCATCCAGGCT-3′.

#### Y2H system construction

We constructed the Y2H for FXR by yeast co-transformation with pGBKT7-FXR LBD (BD) and pGADT7-SRC-1 (AD) according to the lithium acetate method. Briefly, 500 ng of BD and AD were added to 50 μL of the yeast competent cells and mixed with 36 μL of lithium acetate, 240 μL of 50% PEG3350, and 50 ng single-strain DNA at 30°C for 30 min, followed by heat-shock (250 rpm) at 42°C for 30 min. The mixture was subsequently spread on a drop-out-agar plate without leucine and tryptophan (6.7 g/L yeast nitrogen base without amino acids, 1.54 g/L -Leu/-Trp DO supplement, 20 g/L glucose, 20 g/L agar). The plates were incubated at 30°C for 48 h for yeast growth and the PCR method was used to confirm the successful transformation.

#### Y2H assay

We performed Y2H assay to determine the agonistic or antagonistic activities of the compounds. Yeast transformations were incubated with either a control vehicle (DMSO) or the indicated compounds for 24 h in an hFXR agonist testing, and in antagonist assays treated with tested compounds plus 10 μM CDCA. The quantitative α-galactosidase activity assays were carried out by using PNP-α-Gal as the substrate according to the Clontech manual. Each experiment was repeated three times independently.

1. Vortex the overnight culture tube for 0.5–1 min to disperse cell clumps and then each sample needs 200 μL to record the exact OD_600_.
2. Centrifuge the tube at 14,000 rpm (10,000 × g) for 30 s. Incubate 16 μL cell culture medium supernatant with 48 μL PNP-α-Gal at 30°C for 60 min. Be sure to cover microtiter plates with a lid or parafilm to prevent evaporation.
3. Terminate the reaction by adding 136 μL of 1 μM sodium carbonate. Record the optical density of each sample at 410 nm.

The α-galactosidase activity was calculated according to the following formula:

$$\begin{array}{l} \alpha -\text{galactosidase activity}[\text{milliunits}/(\text{mL}\times \text{cell})]\\ \text{​ ​​​}=\frac{OD_{410}\times V_{f}\times 1000}{(\varepsilon \times b)\times t\times V_{i}\times OD_{600}} \end{array}$$

where *t* is the elapsed time of incubation (min), *V*_*f*_ is the final volume of assay (200 μL), *V*_*i*_ is the volume of culture medium supernatant added (16 μL), OD_600_ is the optical density of overnight culture, and ε × *b* is the *p*-nitrophenol molar absorptivity at 410 nm × the light path (cm) = 10.5 mL/μmol (Yeast Protocols Handbook PT3024-1, Clontech).

The agonistic activation and inhibition rates (%) were calculated as follows:

$$\begin{array}{l} \text{agonistic activation}=\frac{GA_{treated}}{GA_{DMSO}}\\ \text{inhibition rate}(\%)=\frac{GA_{CDCA}-GA_{treated}}{GA_{CDCA}-GA_{DMSO}} \end{array}$$

where *GA* indicates α-galactosidase activity.

### Chemistry

The NPD is our in-house collection of over 4,000 natural products isolated from about 100 plants and their structures were established by extensive spectroscopic. The purities of all compounds were checked by using NMR and HPLC (purities ≥ 95%). The detailed data of the natural products mentioned in the report are listed as follows.

**1a**: Abiesatrine B, isolated from *Abies georgei*; amorphous powder; ESI-MS: *m/z* 491 [M + Na]^+^; ^1^H-NMR (600 MHz, CD_3_OD, δ): 2.28 (m), 1.83 (m), 1.95 (m), 1.58 (m), 3.39 (m), 1.45 (m), 1.92 (m), 5.66 (m), 1.42 (m), 2.25 (m), 1.81 (dd, *J* = 14.7, 2.4 Hz), 5.56 (dd, *J* = 8.4, 2.4 Hz), 1.45 (m), 1.92 (m), 0.96 (s), 0.95 (s), 2.21 (m), 0.88 (d, *J* = 6.3 Hz), 2.92 (dd, *J* = 14.1, 1.8 Hz), 2.23 (m), 6.86 (d, *J* = 1.5 Hz), 2.16 (d, *J* = 1.5 Hz), 0.94 (s), 0.92 (s), 1.20 (s). ^13^C-NMR (150 MHz, CD_3_OD, δ): 30.7, 26.5, 77.2, 38.0, 39.4, 24.3, 120.1, 147.5, 52.6, 36.0, 29.1, 123.8, 157.4, 51.2, 37.9, 39.2, 47.6, 25.4, 22.8, 40.2, 16.2, 49.4, 205.5, 129.7, 149.1, 15.9, 174.6, 28.9, 23.6, 26.6.

**1b**: (24Z)-3,23-Dioxo-9βH-lanosta-7,24-dien-27-oic acid, isolated from *Abies georgei*; amorphous powder; ESI-MS: *m/z* 467 [M - H]^−^; ^1^H-NMR (300 MHz, CD_3_OD, δ): 5.67 (1H, dt, *J* = 7.5, 2.7 Hz), 1.94 (3H, d, *J* = 1.2 Hz), 1.08 (3H, s), 1.07 (3H, s), 1.05(3H, s), 0.99 (3H, s), 0.95 (3H, d, *J* = 6.0 Hz), 0.82 (3H, s); ^13^C-NMR (75 MHz, CD_3_OD, δ): 35.2, 35.3, 221.7, 48.1, 53.7, 24.0, 122.8, 149.9, 46.8, 37.0, 21.9, 35.6, 45.2, 53.2, 34.2, 29.5, 54.7, 22.9, 23.5, 34.2, 20.8, 50.1, 200.0, 128.1, 150.2, 173.6, 28.4, 21.7, 27.9.

**1c**: Abiesatrine D, isolated from *Abies georgei*; amorphous powder; ESI-MS: *m/z* 477 [M + H]^+^; ^1^H-NMR (600 MHz, CD_3_OD, δ): 1.73 (m), 1.61(m), 2.50 (dt, *J* = 7.5, 1.8 Hz), 1.42 (dt, *J* = 12.0, 1.2 Hz), 1.94 (m), 1.89 (m), 5.65 (dt, *J* = 7.8, 2.7 Hz), 2.21 (m), 1.64 (m), 1.85 (m), 1.72 (m), 1.60 (m), 1.43 (m), 1.96 (m), 1.29 (m), 1.54 (m), 0.79 (m), 1.00 (s), 1.42 (m), 0.92 (d, *J* = 6.6 Hz), 1.63 (m), 1.58 (m), 2.24 (m), 2.15 (m), 6.19 (dt, *J* = 7.5, 1.2 Hz), 1.85 (brs), 1.10 (s), 1.11 (s), 1.03 (s). ^13^C-NMR (150 MHz, CD_3_OD, δ): 34.2, 34.3, 219.1, 47.0, 52.4, 23.0, 121.5, 148.7, 45.5, 35.8, 20.9, 34.4, 44.0, 51.9, 33.1, 29.7, 53.0, 22.4, 23.1, 36.1, 18.2, 34.6, 26.0, 145.7, 126.6, 172.6, 12.0, 28.0, 21.3, 27.4.

**2a**: 15-Hydroxy-7-oxo-8,11,13-abietatrien-18-oic acid, isolated from *Abies georgei*; amorphous powder; ESI-MS: *m/z* 329 [M - H]^−^; ^1^H-NMR (300 MHz, CD_3_OD, δ): 8.06 (1H, d, *J* = 2.1 Hz), 7.72 (1H, d, *J* = 8.4, 2.1 Hz), 7.43 (1H, d, *J* = 8.4 Hz), 1.51 (6H, s), 1.31 (3H, s), 1.27 (3H, s); ^13^C-NMR (75 MHz, CD_3_OD, δ): 39.1, 19.5, 38.2, 48.2, 45.7, 38.6, 201.5, 131.4, 155.9, 38.6, 124.9, 132.2, 149.1, 124.0, 72.6, 31.7, 31.7, 183.4, 17.4, 23.8.

**2b**: 17-Nor-7,15-dion-8,11,13-abietatrien-18-oic acid, isolated from *Abies georgei*; amorphous powder; ESI-MS: *m/z* 313 [M - H]^−^; ^1^H-NMR (600 MHz, CD_3_OD, δ): 2.48 (m), 1.61 (dt, *J* = 6.9, 3.0 Hz), 1.81 (m), 1.80 (m), 2.68 (dd, *J* = 14.2, 3.0 Hz), 2.86 (dd, *J* = 17.6, 14.2 Hz), 7.63 (d, *J* = 8.4 Hz), 8.18 (dd, *J* = 8.4, 2.1 Hz), 8.52 (d, *J* = 2.1 Hz), 2.61 (s), 1.35 (s), 1.32 (s). ^13^C-NMR (150 MHz, CD_3_OD, δ): 38.2, 19.1, 37.8, 47.5, 45.0, 38.8, 199.5, 132.0, 161.8, 39.5, 125.9, 134.5, 136.6, 128.5, 199.3, 26.7, 181.0, 16.9, 23.5.

**3a**: Daphneone, isolated from *Daphne odora* Thunb. var. *marginata*; white powder; ESI-MS: *m/z* 255 [M + H]^+^; ^1^H-NMR (500 MHz, DMSO-d6, δ): 2.92 (2H, t, *J* = 6.0Hz), 1.61 (2H, t, *J* = 3.0 Hz), 1.61 (2H, t, *J* = 3.0 Hz), 2.60 (2H, t, *J* = 6.0 Hz), 7.84 (2H, d, *J* = 9.0 Hz), 6.85 (2H, d, *J* = 9.0 Hz), 6.85 (2H, d, *J* = 9.0 Hz), 7.84 (2H, d, *J* = 9.0 Hz), 7.18 (m), 7.27 (m), 7.18 (m), 7.27 (m), 7.18 (m), 10.28 (s); ^13^C-NMR (125 MHz, DMSO-d6, δ): 23.7, 30.5, 34.9, 37.1, 115.1, 115.1, 128.3, 128.2, 128.1, 125.5, 128.1, 128.2, 130.3, 130.3, 142.0, 161.8, 198.1.

**3b**: Daphneolon, isolated from *Daphne odora* Thunb. var. *marginata*; white powder; EI-MS: *m/z* 270 [M]; ^1^H-NMR (500 MHz, CD_3_OD, δ): 1.80 (2H, m); 2.72 (1H, m); 3.00 (1H, m); 3.08 (1H, m), 3.09 (1H, m), 4.14 (1H, m), 6.80 (2H, d, *J* = 7.0 Hz), 7.11 (1H, m), 7.15 (4H, m), 7.84 (2H, d, *J* = 7.0 Hz); ^13^C-NMR (125 MHz, CD_3_OD, δ): 31.0, 38.3, 44.6, 66.9, 114.3, 124.8, 127.4, 127.5, 128.5, 130.1, 141.5, 161.9, 198.1.

**3c**: Daphnenone, isolated from *Daphne tangutica* Maxim; white powder; EI-MS: m/z 252 [M]^+^; ^13^C-NMR (125MHz, DMSO-d6, δ): 187.3, 125.9, 146.7, 33.5, 33.7, 128.7, 130.9, 115.3, 162.0, 115.3, 130.9, 141.0, 128.3, 128.3, 125.8, 128.3, 128.3.

**3d**: P-coumaric acid, isolated from *Incarvillea mairei* var. *grandiflora* (Wehrhahn) Grierson; white amorpuous powder; ESI-MS: *m/z* 164 [M]^+^; ^1^H NMR (600 MHz, CD_3_OD, δ): 6.32 (1H, d, *J* = 16.0 Hz), 7.37 (1H, d, *J* = 16.0 Hz), 7.35 (2H, d, *J* = 8.5 Hz), 6.77 (2H, d, *J* = 8.5 Hz), 5.0 (4-OH), 6.41 (-H), 6.77 (3-H,5-H), 7.35 (2-H,6-H), 7.37 (7-H), 11.0 (-H); ^13^C-NMR (150 MHz, CD_3_OD, δ): 128.7 (s), 130.1 (d), 116.6 (d), 159.8 (s), 116.6 (d), 130.1 (d), 141.5 (d), 122.6 (d), 171.5 (s).

**3e**: Ethylparaben, isolated from *Aeschynanthus bracteatus*; Colloidal; ESI-MS: *m/z* 175 [M + Na]^+^; ^1^H-NMR (300 MHz, CD_3_OD, δ): 7.97 (1H, d, *J* = 9.0 Hz), 6.87 (1H, d, *J* = 9.0 Hz), 6.87 (1H, d, *J* = 9.0 Hz), 7.97 (1H, d, *J* = 9.0 Hz), 4.35 (2H, d, *J* = 7.2 Hz), 1.26 (3H, s); ^13^C-NMR (75 MHz, CD_3_OD, δ): 123.1, 131.9, 115.1, 159.7, 115.1, 131.9, 162.9, 60.7, 14.4.

## Results and discussions

### Protein structure selection and redocking validation

The binding of proper modulators to LBD is the molecular basis for FXR activation that triggers the conformational change of FXR, the subsequent coactivators association, and the final target genes regulation. Previous studies have shown that FXR can be activated by structurally diverse agonists, and an array of crystal structures of FXR-LBD complexed with agonists have been solved. The agonist-binding pocket is positioned in the interior of the LBD, and agonists with different skeletons display enormously distinct binding features (Maloney et al., 2000; Soisson et al., 2008; Flatt et al., 2009; Jin et al., 2013). Computational studies, along with crystallographic experiments (Xu et al., 2015), support the notion that the agonist-binding pocket can also be occupied by antagonists (Meyer et al., 2005). Given the variability of the binding pockets occupied by distinct modulators, it is necessary to use different FXR crystallographic models in the virtual screening process, in order to maximize the diversity of hit compounds. At the time when we initiated this study, there were in total 27 crystal structures of FXR in complex with different modulators in the Protein Data Bank. After deleting the structures bound with structurally analogous ligands and those without published biological data, 10 unique protein structures were reserved for further analysis.

Protein flexibility has vital influence on ligand recognition, and even subtle protein conformational changes can significantly affect the results of docking simulations (Kitchen et al., 2004). Nevertheless, the receptor is usually held rigid for most docking procedures, including Glide used in this study, to speed up virtual screening of large databases. To compensate for the limitations of rigid protein conformation, as well as to simply the computational simulations, we decided to select two receptors representing dramatically dissimilar conformations to the other reported crystal structures to perform two independent docking calculations. Both the binding site similarity and ligand similarity profiles were taken into consideration for docking model selection. On the one hand, pairwise binding pocket similarities among the 10 structures were calculated using our in-house program PocketShape, which is designed for computational evaluation of the binding site similarity based on pocket shape and property and could be accessed through the webserver SiteMapper (http://lilab.ecust.edu.cn/). Residues within 5Å distance around the ligand were extracted as the binding site for each structure. Typically, two binding sites with a score value over than 0.8 are considered similar. On the other hand, pairwise molecular similarities among the 10 crystallographic ligands were calculated with SciTegic FCFP_4 fingerprints in Pipeline Pilot v7.5, and a Tc cutoff value of 0.6 was set to define similarity.

The binding pocket and ligand similarity values of the 10 unique crystal structures were plotted in Figure S1. Meanwhile, for each model, the average pocket and ligand similarity values were calculated, respectively, to evaluate its uniqueness. Four crystal structures 1OSH, 1OSV, 3OLF, and 4WVD were considered, as all of them have top five minimum average values ranked by either pocket or ligand similarity calculations. Although both the pocket and ligand similarity values of 4WVD scatter in a low range, its ligand ivermectin is a large macrocyclic lactone (Jin et al., 2013), making the ligand binding pocket expand to a volume of 1081 Å^3^ (Dundas et al., 2006), which is likely to cause artificial enrichment of molecules with large sizes and high molecular weights in docking procedure (Verdonk et al., 2004). Therefore, we did not choose 4WVD. The Tc values of the FXR agonist 6-ECDCA in 1OSV to other ligands are relatively low with predominant scattering below 0.2, and its binding pocket also reveals uniqueness with the average similarity value of 0.62. Moreover, 6-ECDCA is the only FDA-approved FXR modulator so far. Therefore, the 1OSV model was first selected for docking simulation (Mi et al., 2003). After thoroughly analyzing the binding interactions, the biological activities against FXR, as well as the X-ray crystal parameters, the second model of 1OSH was preferentially reserved, the ligand of which has a lower Tc value to 6-ECDCA than that of 3OLF (0.13 vs. 0.15).

Alignment of the 6-ECDCA and fexaramine binding sites exhibited substantial differences in both shape and surrounded residues (Figure S2A). And the tremendous dissimilarities of the two ligands in terms of topological structures and induced binding conformations render them extend to non-overlapping space. To evaluate the docking accuracy of Glide, the two cocrystallized ligands were redocked into the active pocket using Glide XP scoring for each structure. Superimposition of the best redocked poses and the experimental structures (Figures S2B,C) gave the RMSD values of 0.53 Å for 6-ECDCA and 0.65 Å for fexaramine, indicating the robustness of Glide in accurately reproducing the bioactive conformations of the ligands for our two docking models. Cross-docking calculations were also carried out where each crystal structure ligand was docked into the binding pocket of the other. The ligand 6-ECDCA could be docked into 1OSH by Glide XP mode, but the predicted binding pose deviated greatly from the crystallized bioactive conformation (Figure S2D). In agreement with the enormous differences of the two binding sites, fexaramine could not be accommodated by the smaller 6-ECDCA-binding site of 1OSV, hence no proposed docking pose was obtained.

### Virtual screening

**Figure 2:**
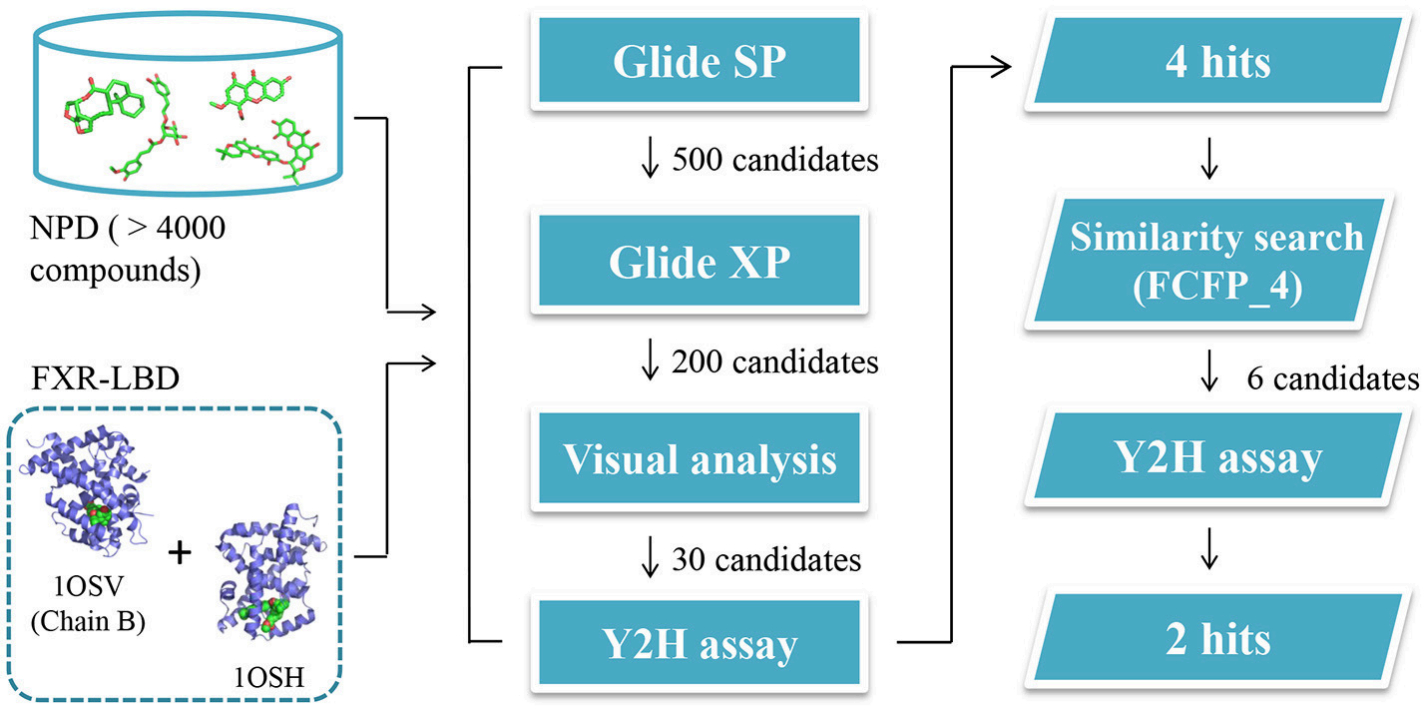
Schematic representation of the whole strategy adopted in this study to identify potential FXR modulators.

To search for potent FXR modulators, structure-based virtual screening strategy, an effective method to identify novel ligands based on predicted binding poses and docking scores, was executed using Glide v6.6 (Maestro v10.1, Schrödinger Inc.). And we speculated that if the docking pose of a certain compound to the agonist-binding pocket was computationally favorable, it could be an effective FXR modulator, either an agonist or an antagonist. The screened natural products database is a collection of over 4000 natural products isolated from about 100 plants, the structures of which have been validated by our researchers. After hierarchical virtual screenings independently implemented with Glide (Figure 2) by using two crystal structures of the receptor, a total of 400 top-ranking compounds were retrieved as candidates from the NPD. These candidates were then subjected to visual inspection to remove those that are likely to be nonbinders. With consideration of key interactions observed from the crystal structures, such as predominant VDW complementarity and some critical hydrogen bonds with polar residues, each docked pose of these candidates was carefully checked to delete the inappropriate compounds. Meanwhile, a specific focus was put on the sizes of the candidates, and those compounds with relatively large groups protruding out of the binding pocket were not considered. Additionally, the compounds with the same scaffold were reserved with a maximum of three to maximize the structural diversity. A total of 30 candidates were finally selected for further bioactivity assay. In the coactivator-recruitment assay based on the Y2H system, none of the 30 compounds could enhance the association of SRC-1 to FXR-LBD, thus no agonist was found. Intriguingly, four compounds (**1a, 2a, 3a, 3c**) strongly inhibited the CDCA-induced SRC-1 recruitment with the inhibition rate higher than 50% in the concentration of 25 μM, displaying apparent FXR antagonistic profiles. The IC_50_ values of the four compounds were determined (Table 1, Figure S4), and guggulsterone was used as the reference compound.

**Table 1:** Chemical structures and activities of FXR antagonists and their analogs reported in this study^a^.

**Compd**.	**Structure**	**Inhibition rate % (25 μM)**	**IC_50_ (μM)^b^**	**Agonistic activation (25 μM)**
**1a**	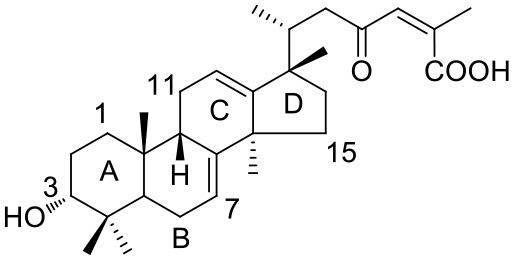	67.35	13.5	1.09
**1b**	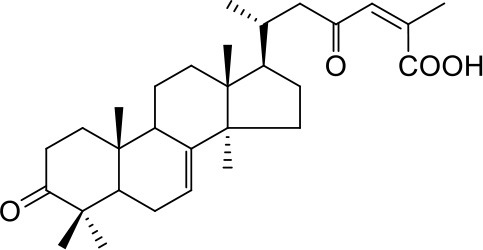	22.66	>25	1.12
**1c**	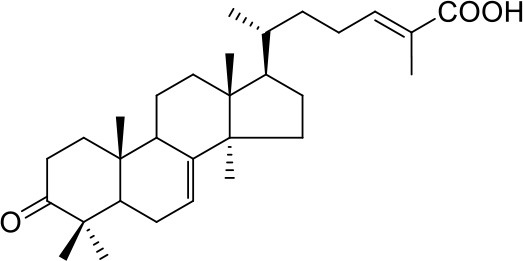	12.84	>25	0.93
**2a**	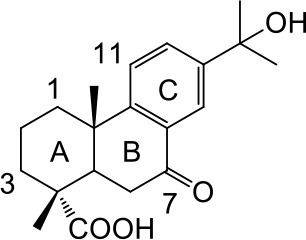	82.16	1.29	1.05
**2b**	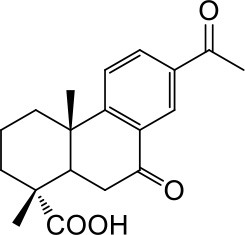	10.05	>25	0.90
**3a**	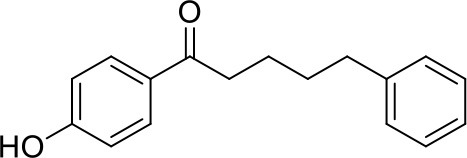	84.45	1.79	1.03
**3b**	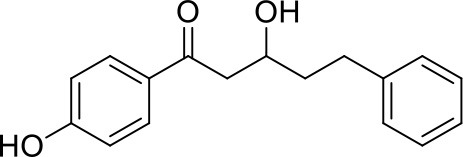	41.4	>25	0.76
**3c**	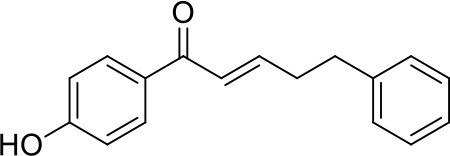	54.9	5.46	1.29
**3d**	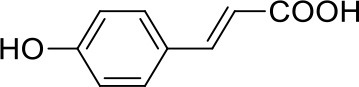	52.8	14.1	1.08
**3e**	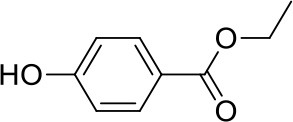	60.1	19.3	0.93
DMSO		0	–	1.00
CDCA		–	–	2.70
Guggulsterone		60.72	6.47	–

a *Data shown are the average values of triplicate measurements determined by Y2H assays. This system employs the interaction between hFXR-LBD and the coactivator SRC-1*.

b *Attempts to determine IC_50_ values were made if the inhibition rate at 25 μM was larger than 50%*.

Starting from the potent FXR antagonists **2a** and **3a** as hit compounds, the in-house NPD was re-screened using similarity searching method, to obtain more potent derivatives as well as to establish underlying structure-activity relationships (SAR). A prior minimum Tc value of 0.6 was first set to retrieve analogous compounds. Unfortunately, only several derivatives were obtained for each hit compound, probably owing to the heterogeneity of the in-house NPD. We, thus, chose a relatively low threshold of 0.3 to maximize the number of obtained analogs. Subsequently, the analogs were also manually checked, and only compounds possessing the same skeleton to the hit compound and with proper sizes were selected. After further *in vitro* Y2H assay, two compounds **3d** and **3e** with the Tc values of 0.4 and 0.41, respectively, to compound **3a**, were found to display moderate antagonistic activities against FXR. As illustrated in Table 1, compounds **3d** and **3e** share the phenol moiety with compound **3a**, and their IC_50_ values were 14.1 and 19.3 μM, respectively.

To evaluate the performance of the virtual screening strategy adopted in this study, the rankings and docking scores of the newly identified natural products were retrospectively examined. The distribution of docking scores of the top ranking candidates reserved from Glide calculations were presented in Figure S3. The natural compounds bearing different scaffolds, including **1c**, **2a**, **2b**, **3b**, and **3c**, could be ranked in the top 500 candidates during the Glide SP docking process using both crystal structures 1OSV and 1OSH, whereas the Glide XP results are totally different. As shown in Table 2, compounds containing similar chemical skeleton to the crystallographic ligand tend to score higher. Moreover, none of the identified FXR antagonists could be simultaneously ranked in the top 200 candidates by the two Glide XP calculations, confirming the rationality of using two distinct crystal structures for structure-based virtual screening. Despite of the confirmed moderate antagonistic effects against FXR, compounds **3d** and **3e** with smaller sizes were excluded after the initial Glide SP screening, which may be ascribed to the recognized bias of structure-based virtual screening method toward high molecular weight compounds (Pan et al., 2003). The results also demonstrate that 2D molecular similarity search method is a powerful and complementary approach to structure-based virtual screening, which could retrieve biologically active compounds that are regarded as false-negatives by docking simulations.

**Table 2:** The rankings and docking scores of the natural products.

**Compd.^a^**	**Glide SP (kcal/mol)**				**Glide XP (kcal/mol)**			
	**1OSV**		**1OSH**		**1OSV**		**1OSH**	
	**Rank**	**Score**	**Rank**	**Score**	**Rank**	**Score**	**Rank**	**Score**
**1a**	20	−10.02	−	−	2	−15.1	−	−
**1b**	37	−9.52	−	−	13	−13.2	−	−
**1c**	395	−7.99	309	−8.16	42	−12.01	−	−
**2a**	59	−9.16	292	−8.23	70	−11.41	−	−
**2b**	93	−8.91	381	−7.87	171	−10.34	−	−
**3a**	−	−	177	−8.61	−	−	142	−10.55
**3b**	488	−7.83	120	−8.85	−	−	139	−10.57
**3c**	390	−8.00	133	−8.80	−	−	195	−10.05
3d*	−	−	−	−	−	−	−	−
3e*	−	−	−	−	−	−	−	−

a Compounds that were ruled out by structure-based virtual screening process but recovered using similarity searching method are labeled with *.

### Structural novelty assessment

**Figure 3:**
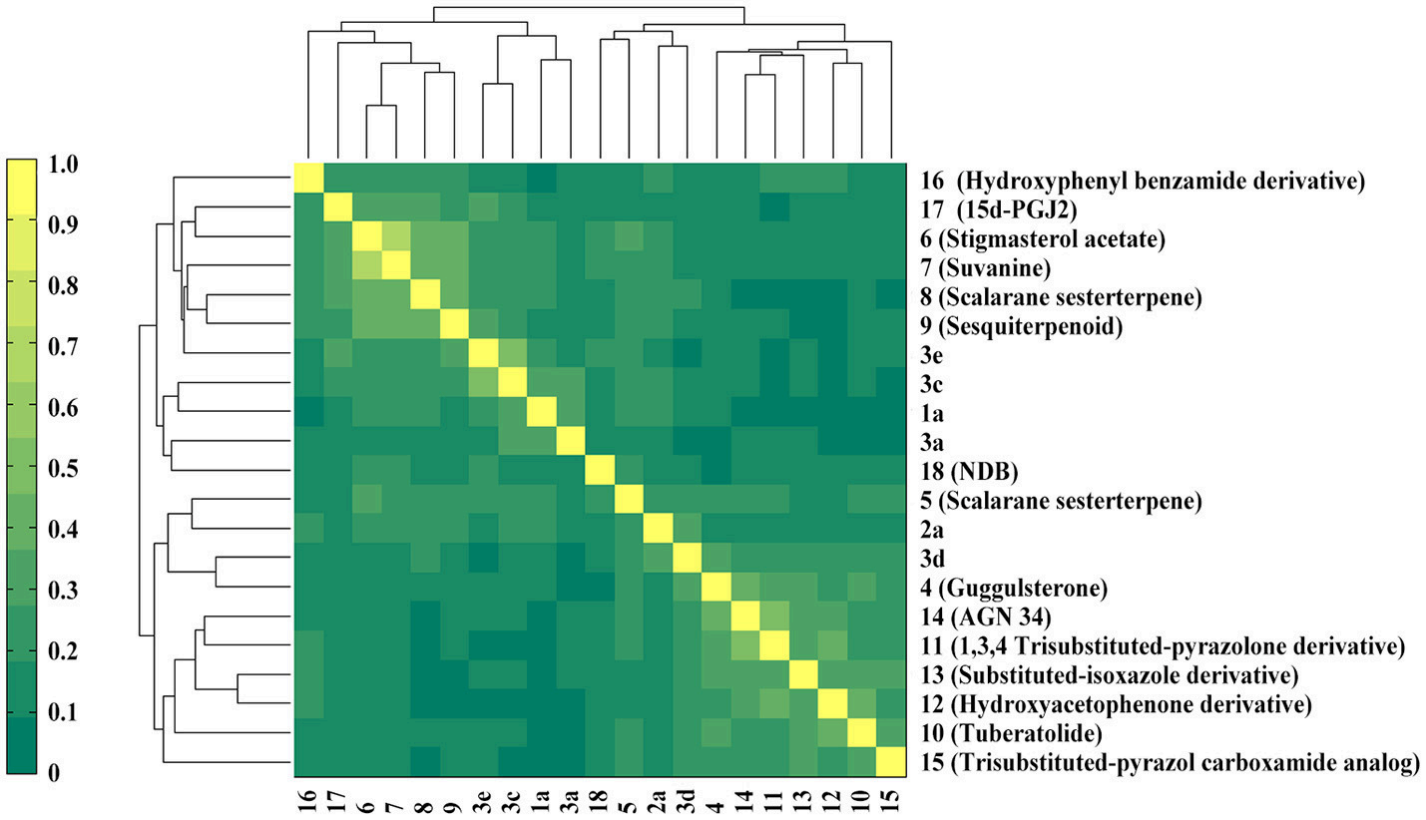
Heatmap presentation of topological similarities of the six natural products to the 15 reported FXR antagonists.

The six natural products were first reported to show FXR antagonistic activity. To evaluate their structural novelty with respect to known FXR antagonists, the pairwise Tc values of chemical similarity were calculated based on the FCFP_4 fingerprints. The Tc value between the similar compounds **3a** and **3c** is 0.67, a Tc cutoff value of 0.6 was therefore set to define similarity. 15 structurally diverse FXR antagonists including seven natural products (compounds **4**–**21**, Table S1) were collected from literatures, among which the maximum Tc value is 0.46. As shown in Figure 3, all the Tc values of the six newly identified hits to the known 15 FXR antagonists were below 0.4, and the maximum Tc values of compounds **1a**, **2a**, and **3a** were 0.38, 0.25, and 0.31 (Table S2), respectively. Accordingly, the six natural products could be considered to be structurally novel as FXR antagonists.

### Analysis of predicted binding poses

From the structural point of view, the six antagonists can be simply categorized into two classes: terpenes possessing polycyclic skeletons and phenols with chain scaffolds. In order to better delineate SAR, their inactive analogs were also displayed and analyzed in this study.

#### Terpenes

**Figure 4:**
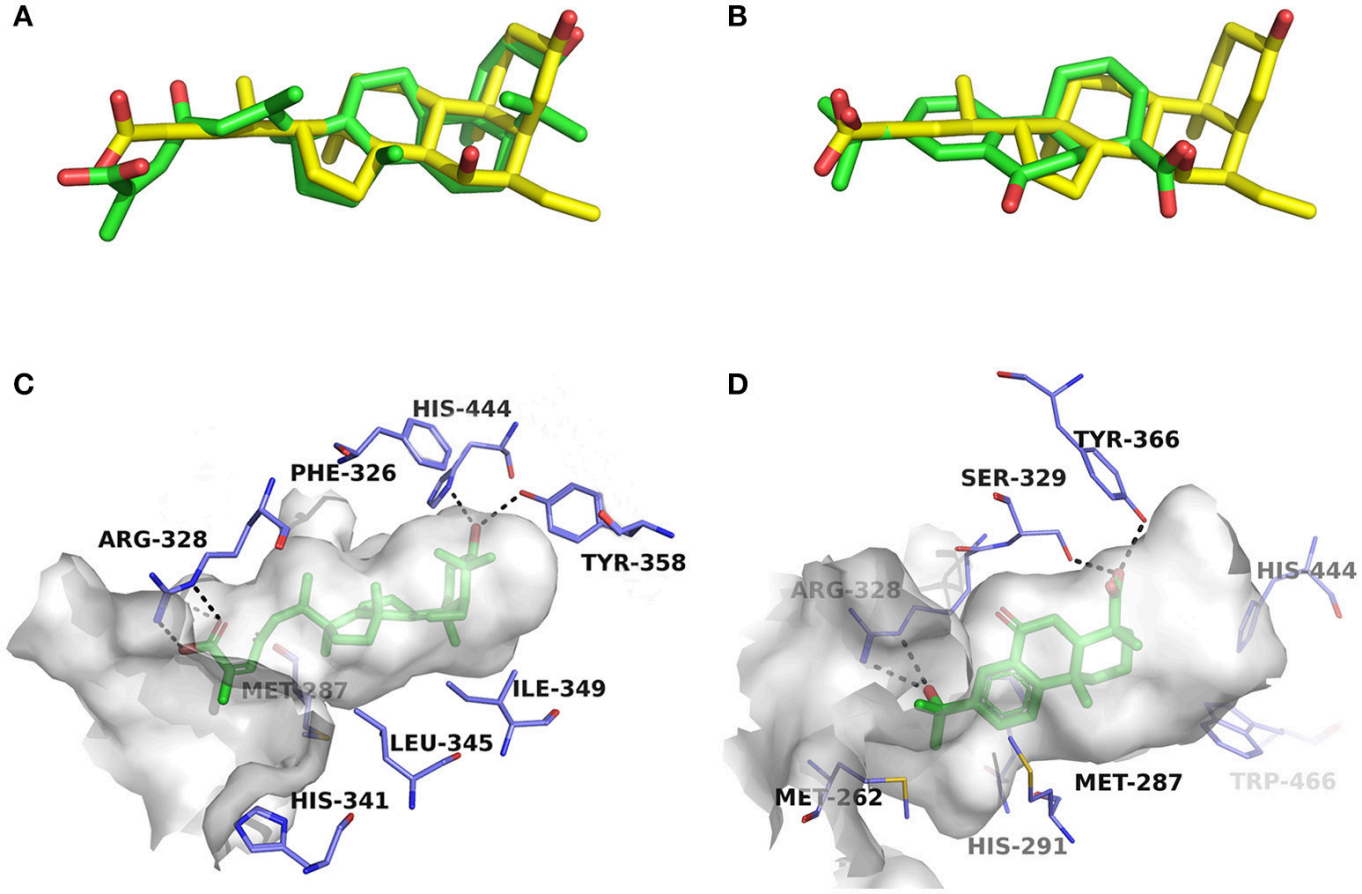
Superposition of docking poses of compounds **1a** and **2a** onto 6-ECDCA **(A,B)** and their proposed interactions with FXR **(C,D)**. The conformation of 6-ECDCA (yellow sticks) was extracted from the crystal structure 1OSV. Compounds **1a** and **2a** are shown as green sticks and hydrogen bonds are highlighted as black dashes. Key residues around the binding pocket are shown as blue lines.

Compounds **1a** and **2a** belong to triterpenes and diterpenes, respectively (Yang et al., 2010), and both of them are isolated from *Abies georgei* which grows exclusively in China. In a previous study, compound **1a** was reported to have moderate agonistic effect against estrogen receptor (ER). The polycyclic ring skeletons of the two compounds are much similar to that of the bile acids, especially for the tetracyclic triterpene compound **1a**. However, the Tc values between compounds **1a** and **2a** and 6-ECDCA are as low as 0.36 and 0.28, respectively. Intriguingly, despite the opposite activities against FXR, the proposed binding poses of the two compounds closely resemble that of 6-ECDCA when interacting with FXR (Figure 4). Similar to 6-ECDCA, compound **1a** adopts *cis*-orientation in the A/B rings linkage, which is considered to be a unique feature for bile acids. The ring skeleton fits the binding pocket well through favorable VDW contacts and hydrophobic effects with adjacent residues. The 3α-OH group extends to the space between helix 7 and helix 10/11 and putatively participates in hydrogen bond interactions with residues Tyr358 and His444. Additionally, the terminal carboxyl group could interact with residue Arg328, located at the entrance of the binding pocket, through salt bridge or hydrogen bond interactions. All the interactions described above are beneficial to the binding of compound **1a** to FXR. However, the hydrogen bond formed between the 7α-OH of 6-ECDCA and Tyr366 is absent for compound **1a** due to its structural variation on ring B. Under the physical-shape discrimination mechanism employed by FXR (Mi et al., 2003), bile acids without a 7α-OH group, such as LCA and DCA, showed extremely weak affinity with FXR, which may also cause the moderate antagonistic activity of compound **1a** against FXR.

For the analogs where the 3α-OH groups are replaced by carbonyl groups (compounds **1b** and **1c**), no detectable antagonistic activity was found in the coactivator recruitment assay. The planarity of the double bond may restrict the carbonyl oxygen atom to a position distant from residues Tyr358 and His444, and the absence of corresponding hydrogen bond interactions presumably results in the loss of agonistic effects against FXR.

Compared with compound **1a**, compound **2a** has a relatively smaller volume, but displayed 10-fold stronger antagonistic activity. Apparently, compound **2a** doesn't fit the canonical mechanism that nuclear receptors' antagonists are usually voluminous than agonists (Meyer et al., 2005). In the proposed binding pose with FXR, compound **2a** reveals the same amphipathic properties as the bile acid ligands. The oxygen atom of the carboxyl group putatively forms hydrogen bond interactions with residues Ser329 and Tyr366. At the other end of compound **2a**, hydrogen bond interactions could also form between the hydroxyl group and Arg328. The hydroxyl group seems to be essential for the antagonistic activity of compound **2a**, as its analog with an acetyl group (compound **2b**) exhibited no observed activity against FXR. Owing to the methyl group located at the10α-position, the carboxyl-substituted hydrocarbon ring A protrudes from the benzene ring panel, making VDW contacts and hydrophobic effects with Met325 on helix 5. Besides, the sequential ring structure could interact with loop H1-H2 (Met262), helix 3 (His291 and Met287), and helix 6 (Leu345) by favorable hydrophobic and VDW interactions.

Because of the relatively smaller volume, compound **2a** is not able to extend to the pocket that is occupied by rings A and B of 6-ECDCA, hence no direct interactions with helix 10/11 and helix 12 were observed. Previous studies have suggested that the π-cation interaction between His444 on helix 10/11 and Trp466 on helix 12 plays a critical role for the active conformation of helix 12 induced by endogenous bile acids (Mi et al., 2003; Pellicciari et al., 2005). Steroid agonists with 3α-OH group could facilitate the π-cation interaction by providing appropriate disposition of His444 through the steric restriction of hydrogen bonds formed between 3α-OH and residues His444 and Tyr358. Consequently, without the ability of establishing the triad of Tyr358, His444, and Trp466, compound **2a** couldn't secure helix 12 in the active conformation, thus preventing the recruitment of coactivators.

#### Phenols

**Figure 5:**
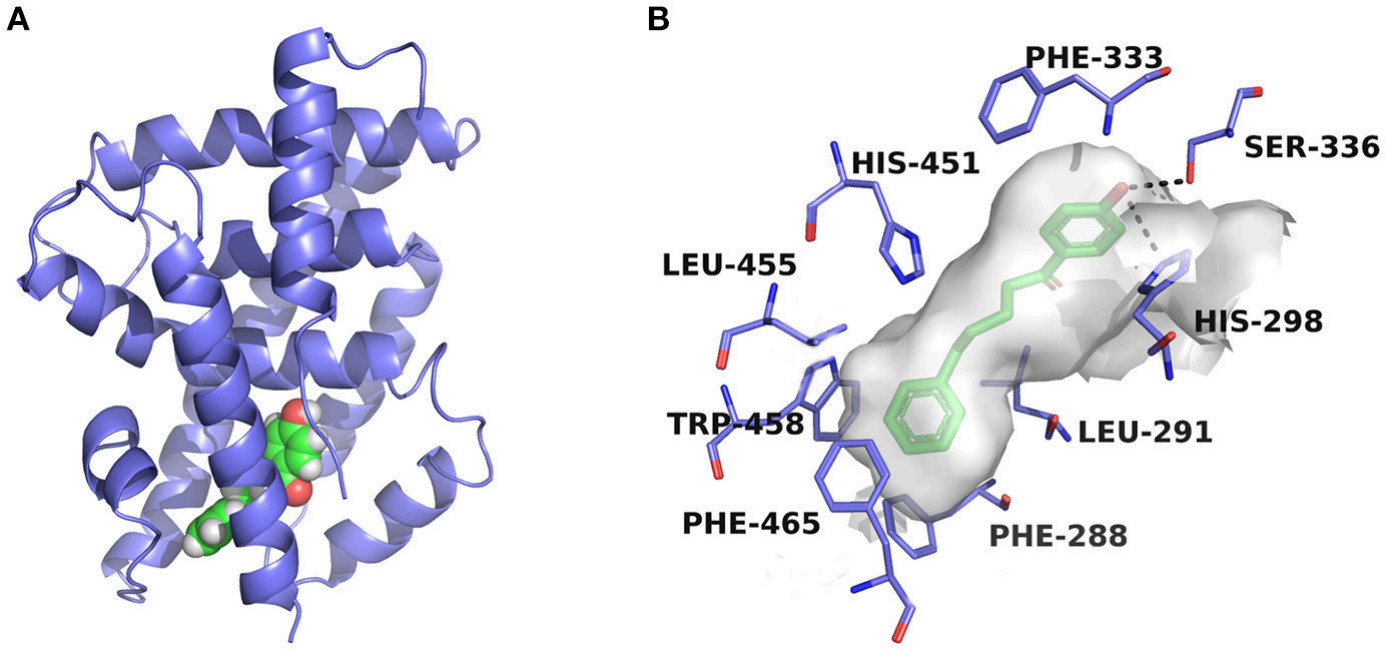
Predicted binding pose of compound **3a** against FXR. **(A)** Overall view. The X-ray crystal structure of the FXR-LBD (PDB ID: 1OSH) is shown in cartoon, and the docked inhibitor is represented by spheres. **(B)** Detailed binding interactions of compound **3a** with FXR. Key residues around the binding pocket are displayed as green lines, and the hydrogen bonds are presented as black dashed lines.

Compounds **3a** and **3c**, named daphneone and daphnenone respectively (Zhang et al., 2006), are constituents of *Daphne odora* Thunb. var. *marginata*, an ornamental plant whose growth is restricted to the south of China. The two compounds, together with their simple structures and small volume, are extraordinarily peculiar to present antagonistic effects against the CDCA-induced SRC-1 association with FXR. Moreover, it is difficult to find common structural features between the two compounds and known agonists or antagonists. Accordingly, we turned to the initial docking poses to probe the structural basis of the FXR antagonistic profiles of this chemical series (Figure 5).

The two compounds were selected from the virtual screening process using the crystal structure of FXR-fexaramine complex. Compound **3a** is sandwiched in the cleft enclosed by helices 5, 7, and 10/11, partially overlapping with the fexaramine-binding pocket. The hydroxyl group points toward helix 5 and may form hydrogen bonds with residues Ser336 and His298. On the other terminal, the benzene ring moiety extends to the aromatic residues-rich groove formed by Phe288 (helix 7), Trp458 (helix 10/11), and Phe465 (loop H10/11-H12), and probably contacts with these residues by advantageous π-stacking interactions. The linker between the two benzene rings fits the pocket by hydrophobic interactions and VDW contacts with surrounding residues such as Leu291, Met294, Ile356, and Ile361.

Previous molecular dynamics simulation studies assumed that the intrinsically unstable loop H10/11-12 controlled the flexibility of helix 12 (Costantino et al., 2005). Through offset face-to-face π-stacking interaction between the benzene ring and Phe465, both compounds **3a** and **3c** could contact with the loop directly, which may interfere with the conformation of the loop and push helix 12 away from its active conformation. For compound **3c** which has a double bond in the linker region, the antagonistic effect in the SRC-1-recruinment assay is slightly weaker. Presumably, the relatively flexible hydrocarbon linker is more suitable for the binding process with FXR-LBD, hence compound **3a** displayed stronger antagonistic activity. When a hydroxyl group was introduced into the hydrocarbon linker, the antagonistic activity of compound **3b** markedly decreased, displaying an IC_50_ value higher than 25 μM. Whereas the much smaller compounds **3d** and **3e**, which share the phenolic moiety with **3a**, exhibited moderate antagonistic effects against FXR. Compounds **3d** and **3e** were docked to FXR by Glide XP mode using the crystal structure 1OSH. As illustrated in Figure S5, the two small molecules occupy merely a fraction of the fexeramine-binding pocket. Hydrophobic effects and shape complementarities presumably dominate the interactions with FXR, as no hydrogen bond was detected in their proposed binding poses.

Notably, compounds **3a** and **3c** have been previously reported to show cytotoxic activities against a variety of human tumor cell lines, including K562, A549, MCF-7, LOVO, HepG2, and A375-S2, with the IC_50_ values ranging from 3.12 to 51.0 μM (Zhang et al., 2006; Wang et al., 2012). Besides, the agonistic profiles of compounds **3d** and **3e** against ER have also been described in a previous study (Cao et al., 2013). The phenol FXR antagonists identified in this study are relatively small, especially for compounds **3d** and **3e**, which probably have effects on other targets in living cells. Further thorough investigations are ongoing to better elucidate the exact mechanisms of action of the newly identified natural FXR antagonists and their implications regarding *in vivo* pharmacological effects.

### Druglikeness evaluation

To assess the drug-like profiles of the six natural products, an *in silico* prediction of ADME properties was performed using QikProp v4.3 module integrated into Maestro 10.1, and 6-ECDCA was used as the reference compound (Table 3). All physically significant descriptors and pharmaceutically relevant properties of the natural FXR antagonists, except for compound **1a**, fall into the recommended ranges of 95% of known drugs, suggesting remarkable potential of druglikeness. The QPlogPo/w and QPlogS values of compound **1a** exceed the limits of either Lipinski's rule of five or Jorgensen's rule of three, therefore the aqueous/lipid solubility should be taken into consideration if further structural optimization was carried out based on the tetracyclic triterpene compound **1a**.

**Table 3:** *In silico* predicted properties of the six FXR antagonists.

**Compd**.	**MW^a^**	**donorHB^b^**	**accptHB^c^**	**#rotor^d^**	**QPlogS^e^**	**QPlogPo/w^f^**	**QPPCaco^g^**	**Rule of five^h^**	**Rule of three^i^**
**1a**	468.67	2.0	5.7	7	−6.60	5.52	142.21	1	1
**2a**	330.42	2.0	4.75	3	−4.23	3.03	82.62	0	0
**3a**	254.32	1.0	2.75	7	−4.27	3.92	1315.91	0	0
**3c**	252.31	1.0	2.75	6	−4.33	3.59	1160.27	0	0
**3d**	164.16	2.0	2.75	4	−1.55	1.38	68.09	0	0
**3e**	166.17	1.0	2.75	3	−2.27	2.46	1083.37	0	0
6-ECDCA	420.63	3.0	5.40	7	−5.36	4.28	48.73	0	0

The recommended ranges by QikProp are as follows:

a *Molecular weight, 130.0–725.0*.

b *Number of hydrogen bond donors, 0.0–6.0*.

c *Number of hydrogen bond acceptors, 2.0–20.0*.

d *Number of non-trivial rotatable bonds, 0–15*.

e *Predicted aqueous solubility, −6.5–0.5*.

f *Predicted octanol/water partition coefficient, −2.0–6.5*.

g *Predicted apparent Caco-2 cell permeability in nm/sec, <25 poor, >500 great*.

h *Number of violations of Lipinski's rule of five, maximum is 4*.

i *Number of violations of Jorgensen's rule of three, maximum is 3*.

## Conclusion

In summary, we have established a small NPD containing over 4,000 compounds that were previously isolated from about 100 medicinal plants. From the database, six FXR antagonists were identified by strategic virtual screening method, which validated the feasibility of virtual screening to explore the potential targets of natural products. Although procured on the basis of known agonist-binding pocket, two of the most potent compounds **2a** and **3a** could antagonize the CDCA-induced SRC-1 recruitment to FXR-LBD with the IC_50_ values of 1.29 μM and 1.79 μM, respectively. The predicted docking mode of the diterpene **2a** against FXR exhibited partially similar binding interactions to those of the crystallographic ligand 6-ECDCA bound to FXR, whereas the daphneone **3a** showed noncanonical proposed binding mode, which may directly contact with the intrinsically unstable loop H10/11-12 by π-stacking interactions with the aromatic residue Phe465. Moreover, as assessed by QikProp, most of the natural FXR antagonists displayed comparable drug-like properties to that of 95% of known drugs. We hope our discovery will provide promising chemical scaffolds for further hit-to-lead optimization and for the study of FXR-related biological mechanisms.

## Author contributions

HL, WZ, and LS conceived the study. YD and SL performed molecular simulations. YD analysed the data and drafted the manuscript. JJ, SZ, and JH carried out experimental studies. HL reviewed and revised the manuscript. All authors have read and approved the final manuscript.

### Conflict of interest statement

The authors declare that the research was conducted in the absence of any commercial or financial relationships that could be construed as a potential conflict of interest.

## References

[B1] Bender A. Jenkins J. L. Scheiber J. Sukuru S. C. Glick M. Davies J. W. (2009). How similar are similarity searching methods? A principal component analysis of molecular descriptor space. J. Chem. Inf. Model. 49, 108–119. 10.1021/ci800249s 19123924

[B2] Cao X. Jiang J. Zhang S. Zhu L. Zou J. Diao Y.. (2013). Discovery of natural estrogen receptor modulators with structure-based virtual screening. Bioorg. Med. Chem. Lett. 23, 3329–3333. 10.1016/j.bmcl.2013.03.105 23608764

[B3] Carotti A. Marinozzi M. Custodi C. Cerra B. Pellicciari R. Gioiello A.. (2014). Beyond bile acids: targeting Farnesoid X Receptor (FXR) with natural and synthetic ligands. Curr. Top. Med. Chem. 14, 2129–2142. 10.2174/1568026614666141112094058 25388537

[B4] Choi H. Hwang H. Chin J. Kim E. Lee J. Nam S. J.. (2011). Tuberatolides, potent FXR antagonists from the Korean marine tunicate botryllus tuberatus. J. Nat. Prod. 74, 90–94. 10.1021/np100489u 21142112

[B5] Costantino G. Entrena-Guadix A. Macchiarulo A. Gioiello A. Pellicciari R. (2005). Molecular dynamics simulation of the ligand binding domain of farnesoid X receptor. Insights into helix-12 stability and coactivator peptide stabilization in response to agonist binding. J. Med. Chem. 48, 3251–3259. 10.1021/jm049182o 15857131

[B6] Downes M. Verdecia M. A. Roecker A. J. Hughes R. Hogenesch J. B. Kast-Woelbern H. R.. (2003). A chemical, genetic, and structural analysis of the nuclear bile acid receptor FXR. Mol. Cell 11, 1079–1092. 10.1016/S1097-2765(03)00104-7 12718892 PMC6179153

[B7] Dundas J. Ouyang Z. Tseng J. Binkowski A. Turpaz Y. Liang J. (2006). CASTp: computed atlas of surface topography of proteins with structural and topographical mapping of functionally annotated residues. Nucleic Acids Res. 34, W116–W118. 10.1093/nar/gkl282 16844972 PMC1538779

[B8] Dussault I. Beard R. Lin M. Hollister K. Chen J. Xiao J.. (2003). Identification of gene-selective modulators of the bile acid receptor FXR. J. Biol. Chem. 278, 7027–7033. 10.1074/jbc.M209863200 12496277

[B9] Fields S. Sternglanz R. (1994). The two-hybrid system: an assay for protein-protein interactions. Trends Genet. 10, 286–292. 10.1016/0168-9525(90)90012-U 7940758

[B10] Filho C. D. D. Downes M. Evans R. M. (2017). Farnesoid X receptor an emerging target to combat obesity. Digest. Dis. 35, 185–190. 10.1159/000450909 PMC5417073 28249279

[B11] Fiorucci S. Mencarelli A. Distrutti E. Palladino G. Cipriani S. (2010). Targeting farnesoid-X-receptor: from medicinal chemistry to disease treatment. Curr. Med. Chem. 17, 139–159. 10.2174/092986710790112666 19941473

[B12] Flatt B. Martin R. Wang T. L. Mahaney P. Murphy B. Gu X. H.. (2009). Discovery of XL335 (WAY-362450), a highly potent, selective, and orally active agonist of the farnesoid X receptor (FXR). J. Med. Chem. 52, 904–907. 10.1021/jm8014124 19159286

[B13] Forman B. M. Goode E. Chen J. Oro A. E. Bradley D. J. Perlmann T.. (1995). Identification of a nuclear receptor that is activated by farnesol metabolites. Cell 81, 687–693. 10.1016/0092-8674(95)90530-8 7774010

[B14] Friesner R. A. Banks J. L. Murphy R. B. Halgren T. A. Klicic J. J. Mainz D. T.. (2004). Glide: a new approach for rapid, accurate docking and scoring. 1. Method and assessment of docking accuracy. J. Med. Chem. 47, 1739–1749. 10.1021/jm0306430 15027865

[B15] Gaulton A. Hersey A. Nowotka M. Bento A. P. Chambers J. Mendez D.. (2017). The ChEMBL database in 2017. Nucleic Acids Res. 45, D945–D954. 10.1093/nar/gkw1074 27899562 PMC5210557

[B16] Gonzalez F. J. Jiang C. T. Patterson A. D. (2016). An intestinal microbiota-farnesoid X receptor axis modulates metabolic disease. Gastroenterology 151, 845–859. 10.1053/j.gastro.2016.08.057 27639801 PMC5159222

[B17] Halgren T. A. Murphy R. B. Friesner R. A. Beard H. S. Frye L. L. Pollard W. T.. (2004). Glide: a new approach for rapid, accurate docking and scoring. 2. Enrichment factors in database screening. J. Med. Chem. 47, 1750–1759. 10.1021/jm030644s 15027866

[B18] Huang H. Yu Y. Gao Z. Zhang Y. Li C. Xu X.. (2012). Discovery and optimization of 1,3,4-trisubstituted-pyrazolone derivatives as novel, potent, and nonsteroidal farnesoid Xreceptor (FXR) selective antagonists. J. Med. Chem. 55, 7037–7053. 10.1021/jm3002718 22862148

[B19] Jin L. Feng X. Rong H. Pan Z. Inaba Y. Qiu L.. (2013). The antiparasitic drug ivermectin is a novel FXR ligand that regulates metabolism. Nat. Commun. 4:1937. 10.1038/ncomms2924 23728580

[B20] Kitchen D. B. Decornez H. Furr J. R. Bajorath J. (2004). Docking and scoring in virtual screening for drug discovery: methods and applications. Nat. Rev. Drug Discov. 3, 935–949. 10.1038/nrd1549 15520816

[B21] Lee F. Y. Lee H. Hubbert M. L. Edwards P. A. Zhang Y. (2006). FXR, a multipurpose nuclear receptor. Trends Biochem. Sci. 31, 572–580. 10.1016/j.tibs.2006.08.002 16908160

[B22] Lew J. L. Zhao A. Yu J. Huang L. de Pedro N. Pelaez F.. (2004). The farnesoid X receptor controls gene expression in a ligand-and promoter-selective fashion. J. Biol. Chem. 279, 8856–8861. 10.1074/jbc.M306422200 14684751

[B23] Li F. Jiang C. T. Krausz K. W. Li Y. Albert I. Hao H.. (2013). Microbiome remodelling leads to inhibition of intestinal farnesoid X receptor signalling and decreased obesity. Nat. Commun. 4:2384. 10.1038/ncomms3384 24064762 PMC6595219

[B24] Lin J. S. Lai E. M. (2017). Protein-protein interactions: yeast two-hybrid system. Methods Mol. Biol. 1615, 177–187. 10.1007/978-1-4939-7033-9_14 28667612

[B25] Lin Z. Shen H. Huang J. Chen S. Chen L. Chen J.. (2008). Butyl 4-(butyryloxy) benzoate functions as a new selective estrogen receptor [beta] agonist and induces GLUT4 expression in CHO-K1 cells. J. Steroid Biochem. 110, 150–156. 10.1016/j.jsbmb.2008.03.028 18455388

[B26] Maglich J. M. Caravella J. A. Lambert M. H. Willson T. M. Moore J. T. Ramamurthy L. (2003). The first completed genome sequence from a teleost fish (Fugu rubripes) adds significant diversity to the nuclear receptor superfamily. Nucleic Acids Res. 31, 4051–4058. 10.1093/nar/gkg444 12853622 PMC165959

[B27] Makishima M. Okamoto A. Y. Repa J. J. Tu H. Learned R. M. Luk A.. (1999). Identification of a nuclear receptor for bile acids. Science 284, 1362–1365. 10334992 10.1126/science.284.5418.1362

[B28] Maloney P. R. Parks D. J. Haffner C. D. Fivush A. M. Chandra G. Plunket K. D.. (2000). Identification of a chemical tool for the orphan nuclear receptor FXR. J. Med. Chem. 43, 2971–2974. 10.1021/jm0002127 10956205

[B29] Meyer U. Costantino G. Macchiarulo A. Pellicciari R. (2005). Is antagonism of E/Z-guggulsterone at the farnesoid X receptor mediated by a noncanonical binding site? A molecular modeling study. J. Med. Chem. 48, 6948–6955. 10.1021/jm0505056 16250653

[B30] Mi L. Z. Devarakonda S. Harp J. M. Han Q. Pellicciari R. Willson T. M.. (2003). Structural basis for bile acid binding and activation of the nuclear receptor FXR. Mol. Cell 11, 1093–1100. 10.1016/S1097-2765(03)00112-6 12718893

[B31] Nam S. J. Ko H. Ju M. K. Hwang H. Chin J. W. Ham J.. (2007). Scalarane sesterterpenes from a marine sponge of the genus spongia and their FXR antagonistic activity. J. Nat. Prod. 70, 1691–1695. 10.1021/np070024k 17988093

[B32] Nevens F. Andreone P. Mazzella G. Strasser S. I. Bowlus C. Invernizzi P.. (2016). A placebo-controlled trial of obeticholic acid in primary biliary cholangitis. N. Engl. J. Med. 375, 631–643. 10.1056/NEJMoa1509840 27532829

[B33] Pan Y. Huang N. Cho S. MacKerell A. D. Jr. (2003). Consideration of molecular weight during compound selection in virtual target-based database screening. J. Chem. Inf. Comput. Sci. 43, 267–272. 10.1021/ci020055f 12546562

[B34] Pellicciari R. Costantino G. Fiorucci S. (2005). Farnesoid X receptor: from structure to potential clinical applications. J. Med. Chem. 48, 5383–5403. 10.1021/jm0582221 16107136

[B35] Pellicciari R. Fiorucci S. Camaioni E. Clerici C. Costantino G. Maloney P. R. (2002). 6 alpha-ethyl-chenodeoxycholic acid (6-ECDCA), a potent and selective FXR agonist endowed with anticholestatic activity. J. Med. Chem. 45, 3569–3572. 10.1021/jm025529g 12166927

[B36] Schaap F. G. Trauner M. Jansen P. L. (2014). Bile acid receptors as targets for drug development. Nat. Rev. Gastro. Hepat. 11, 55–67. 10.1038/nrgastro.2013.151 23982684

[B37] Soisson S. M. Parthasarathy G. Adams A. D. Sahoo S. Sitlani A. Sparrow C.. (2008). Identification of a potent synthetic FXR agonist with an unexpected mode of binding and activation. Proc. Natl. Acad. Sci. U.S.A. 105, 5337–5342. 10.1073/pnas.0710981105 18391212 PMC2291122

[B38] Urizar N. L. Liverman A. B. Dodds D. T. Silva F. Ordentlich P. Yan Y.. (2002). A natural product that lowers cholesterol as an antagonist ligand for FXR. Science 296, 1703–1706. 10.1126/science.1072891 11988537

[B39] Verdonk M. L. Berdini V. Hartshorn M. J. Mooij W. T. Murray C. W. Taylor R. D.. (2004). Virtual screening using protein-ligand docking: avoiding artificial enrichment. J. Chem. Inf. Comp. Sci. 44, 793–806. 10.1021/ci034289q 15154744

[B40] Wang L. B. Dong N. W. Wu Z. H. Wu L. J. (2012). Two new compounds with cytotoxic activity on the human melanoma A375-S2 cells from Daphne giraldii callus cells. J. Asian Nat. Prod. Res. 14, 1020–1026. 10.1080/10286020.2012.701206 23106433

[B41] Wang Y. D. Chen W. D. Moore D. D. Huang W. (2008). FXR: a metabolic regulator and cell protector. Cell Res. 18, 1087–1095. 10.1038/cr.2008.289 18825165

[B42] Wu J. Xia C. S. Meier J. Li S. Z. Hu X. Lala D. S. (2002). The hypolipidemic natural product guggulsterone acts as an antagonist of the bile acid receptor. Mol. Endocrinol. 16, 1590–1597. 10.1210/mend.16.7.0894 12089353

[B43] Xu X. Xu X. Liu P. Zhu Z. Y. Chen J. Fu H. A. (2015). Structural basis for small molecule NDB (N-benzyl-N-(3-(tert-butyl)-4-hydroxyphenyl)-2,6-dichloro-4-(dimethylamino) benzamide) as a selective antagonist of farnesoid X receptor alpha (FXR alpha) in stabilizing the homodimerization of the receptor. J. Biol. Chem. 290, 19888–19899. 10.1074/jbc.M114.630475 26100621 PMC4528148

[B44] Yamada T. Sugimoto K. (2016). Guggulsterone and its role in chronic diseases. Adv. Exp. Med. Biol. 929, 329–361. 10.1007/978-3-319-41342-6_15 27771932

[B45] Yang X. W. Feng L. Li S. M. Liu X. H. Li Y. L. Wu L. (2010). Isolation, structure, and bioactivities of abiesadines AY, 25 new diterpenes from Abies georgei Orr. Bioorg. Med. Chem. 18, 744–754. 10.1016/j.bmc.2009.11.055 20022253

[B46] Yuan Z. Q. Li K. W. (2016). Role of farnesoid X receptor in cholestasis. J. Digest. Dis. 17, 501–509. 10.1111/1751-2980.12378 27383832

[B47] Zhang W. Zhang W. D. Liu R. H. Shen Y. Zhang C. Cheng H.. (2006). Two new chemical constituents from Daphne odora Thunb. var. marginata. Nat. Prod. Res. 20, 1290–1294. 10.1080/14786410601101860 17393653

[B48] Zhang Y. Edwards P. (2008). FXR signaling in metabolic disease. FEBS Lett. 582, 10–18. 10.1016/j.febslet.2007.11.015 18023284

