# Supplementary material for: Discovery of Natural Products as Novel and Potent FXR Antagonists by Virtual Screening
Source: Front Chem. 2018 Apr 30;6:140. doi: 10.3389/fchem.2018.00140 (PMC5936786; doi:10.3389/fchem.2018.00140)
Supplement: Supplementary file 1 [file Data_Sheet_1.pdf]

Supplementary Material

Discovery of Natural Products as Novel and Potent FXR Antagonists by Virtual Screening

Yanyan Diao [1], Jing Jiang [1], Shoude Zhang [1], Shiliang Li [1], Lei Shan [2], Jin Huang [1], Weidong Zhang [2], and Honglin Li [1,*]

[1] Shanghai Key Laboratory of New Drug Design, State Key Laboratory of Bioreactor Engineering, School of Pharmacy, East China University of Science and Technology, Shanghai 200237, China

[2] Department of Phytochemistry, School of Pharmacy, Second Military Medical University, Shanghai 200433, China

*** Correspondence:**
Prof. Honglin Li
hlli@ecust.edu.cn

Supplementary Figures and Tables

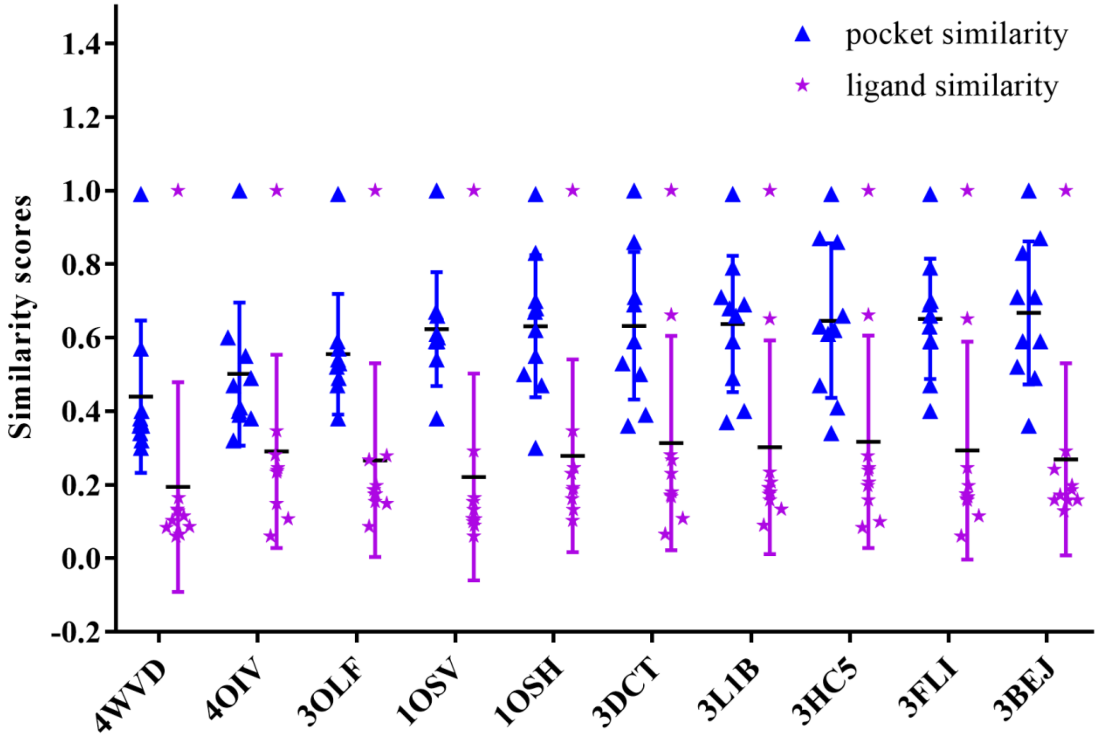

Figure S1. Scatter presentation of pocket and molecule similarity values among the 10 unique FXR crystal structures in complex with structurally distinct modulators. The PDB codes were arranged in ascending order of their average pocket similarity values.

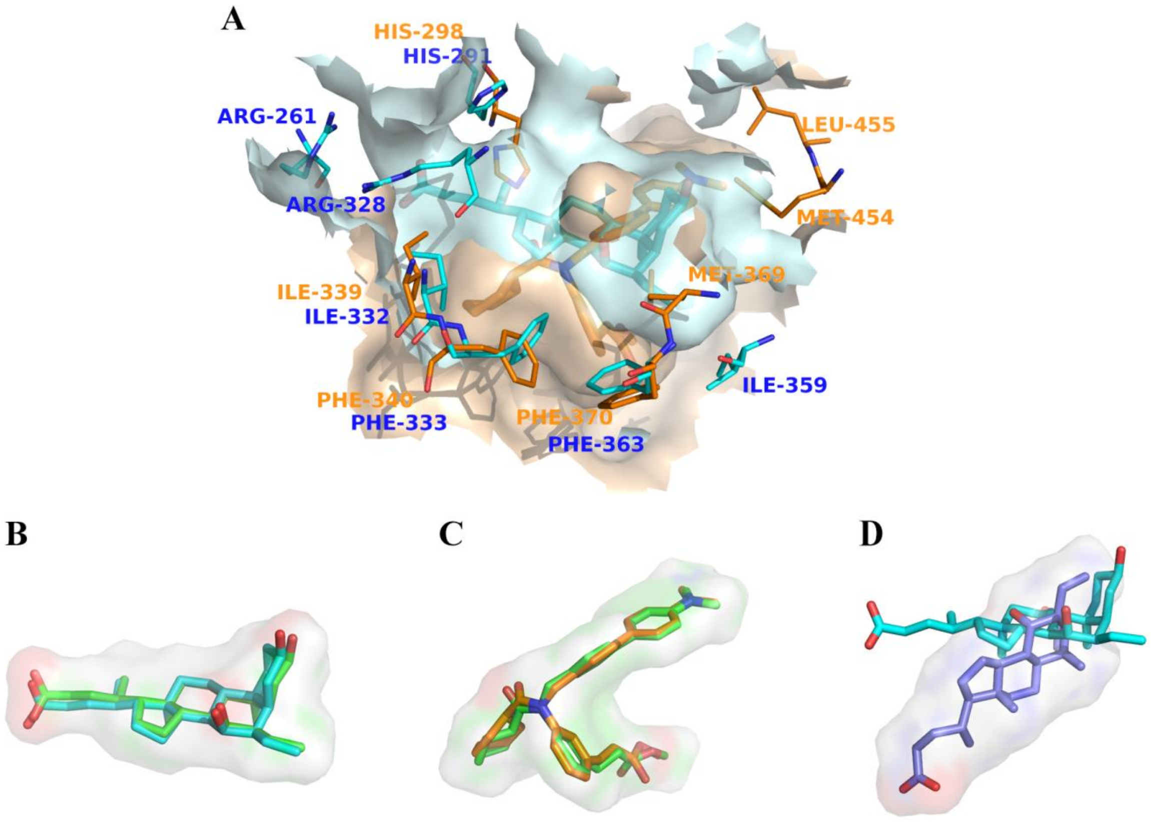

Figure S2. (A) Binding pockets comparison of 1OSV and 1OSH. The pocket surface surrounded 6-ECDCA and fexaramine are colored pale cyan and light orange, respectively, and some residues representing the pockets are hidden for clarity. (B) Redocking result of 1OSV. (C) Redocking result of 1OSH. The best redocked poses are shown as green sticks, and the docking scores are -16.25 kcal/mol and -15.12 kcal/mol, respectively. (D) Crossdocking result of 6-ECDCA (purple) to 1OSH. The docking score is -6.95 kcal/mol. The ligand fexaramine can’t be docked to the 6-ECDCA-binding site in 1OSV, therefore no data was shown.

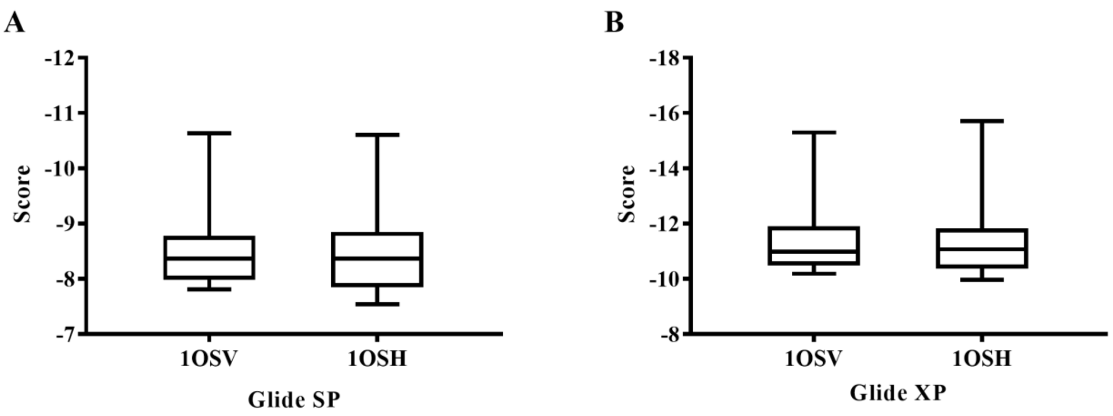

Figure S3. Distribution of docking scores of the top 500 candidates ranked by Glide SP mode (A) and the top 200 candidates ranked by Glide XP mode (B).

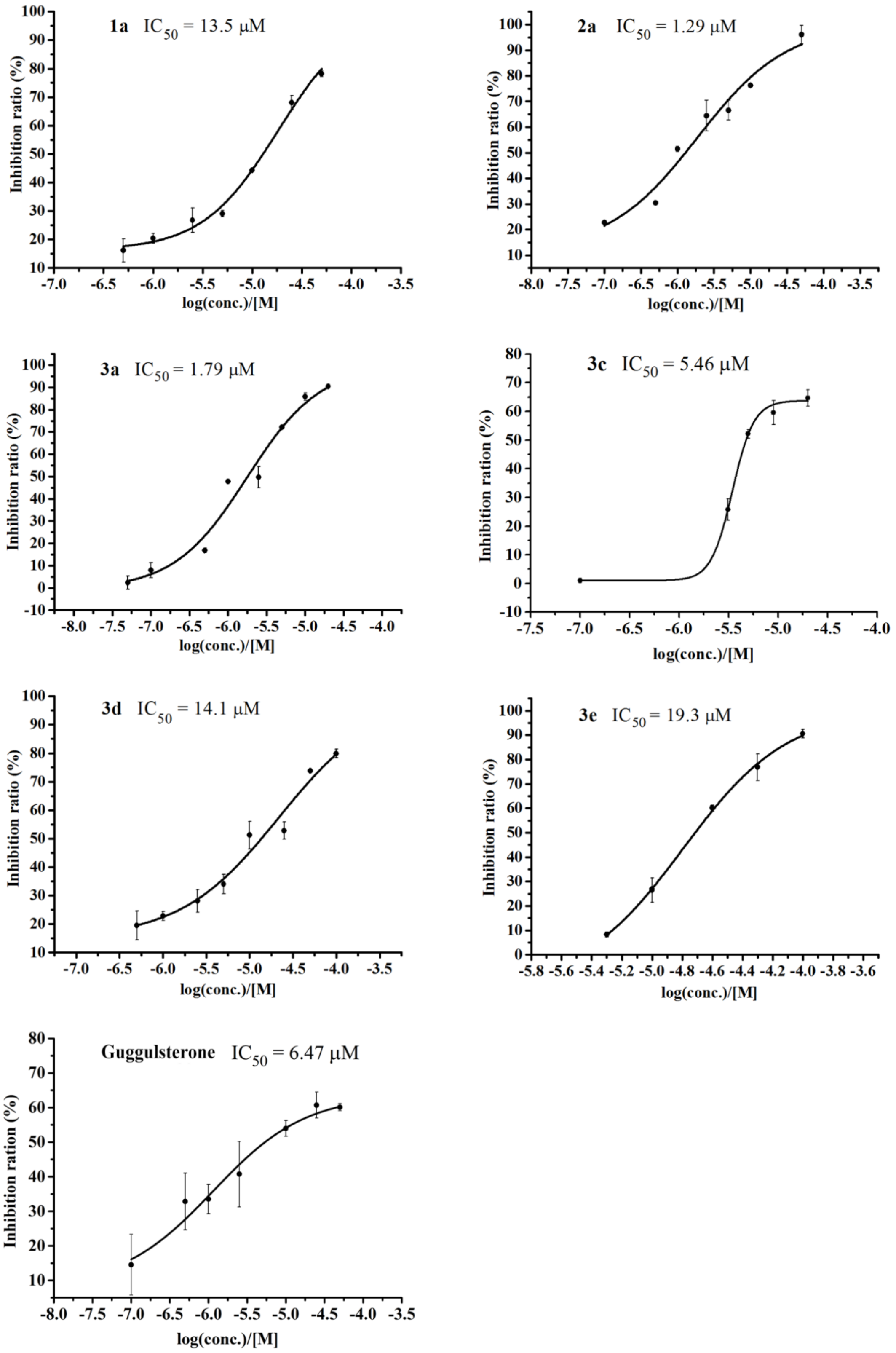

Figure S4. Dose-response curves of FXR antagonists reported in this study and the reference compound guggulsterone.

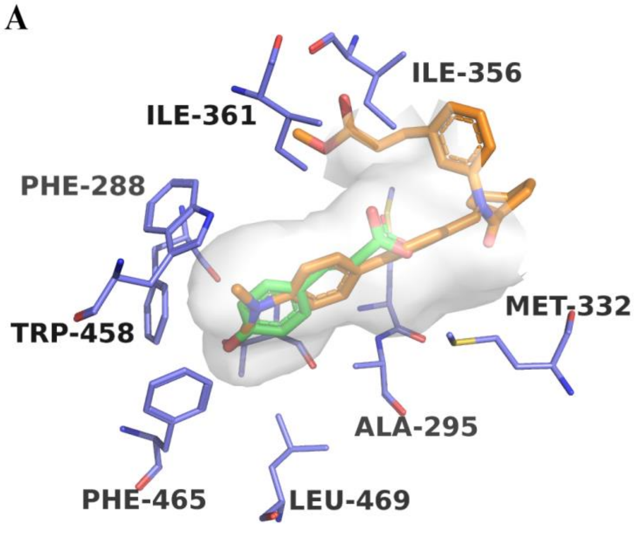

Figure S5. Propose docking poses of compounds **3d** and **3e** (green) in the fexeramine (orange)-binding pocket (PDB code 1OSH). Key residues around the binding pocket are shown as purple lines. The docking scores are -6.50 kcal/mol and -6.15 kcal/mol, respectively.

Table S1. Chemical structures and activities of the 15 FXR antagonists collected from literatures.

No.	Structure	Activity description	Experimental method	Reference
**4**	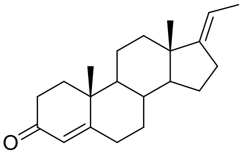	Ki > 5 μM	Radioligand binding assay	(Wu et al., 2002)
**5**	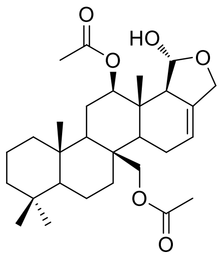	IC_{50} = 8.1 μM	Cotransfection assay	(Nam et al., 2006)
**6**	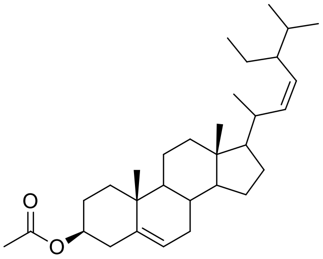	suppress BA-activated, FXR-mediated reporter gene expression	Cell-based transfection assay	(Carter et al., 2007)
**7**	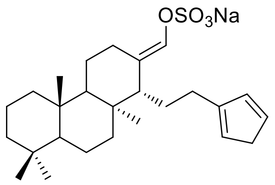	EC_{50} $\approx$ 24 μM.	FXR transactivation assay	(Di Leva et al., 2013)
**8**	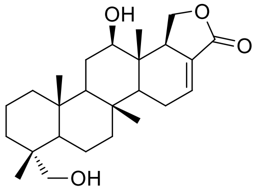	IC_{50} = 2.4 μM	Cotransfection assay	(Nam et al., 2007)
**9**	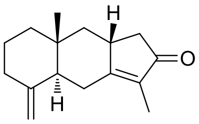	IC_{50} = 50 μM	Transient transfection reporter assay in HepG2 cells	(Tsai et al., 2012)

**10**	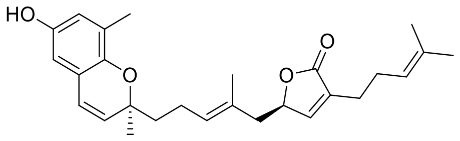	IC_{50} = 1.5 μM	Cotransfection assay	(Choi et al., 2011)
**11**	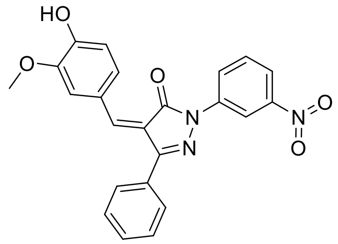	IC_{50} = 8.96 μM	Homogeneous time-resolved fluorescence assay	(Huang et al., 2012)
**12**	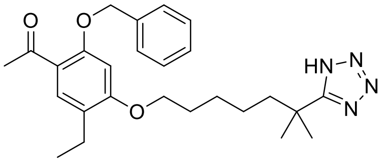	IC_{50} = 1.1 μM	Homogeneous time-resolved fluorescence assay	(Liu et al., 2014)
**13**	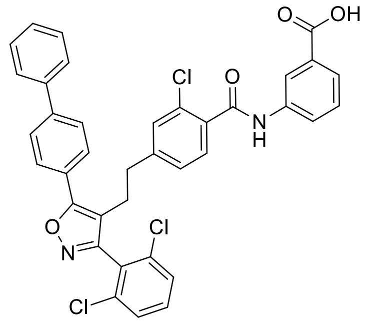	IC_{50} = 3.7 μM	Cotransfection assay	(Kainuma et al., 2007)
**14**	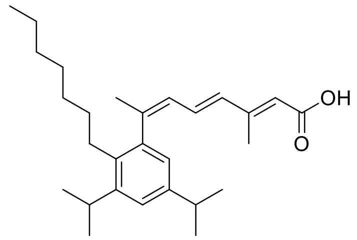	IC_{50} = 0.3 μM	Agonist-induced coactivator recruitment	(Dussault et al., 2003)
**15**	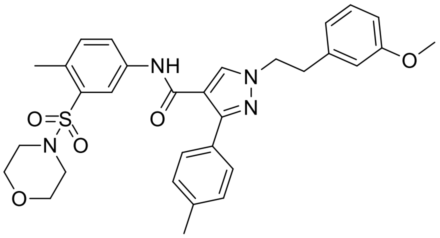	IC_{50} = 0.0075 μM	FXR biochemical TR-FRET binding assay	(Yu et al., 2014)

**16**	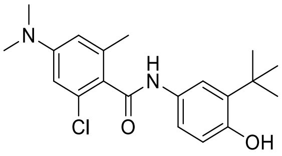	IC_{50} = 1.4 μM	Homogeneous time-resolved fluorescence assay	(Song et al., 2015)
**17**	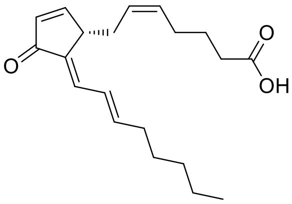	IC_{50} = 7.25 μM	Homogeneous time-resolved fluorescence assay	(Xu et al., 2013)
**18**	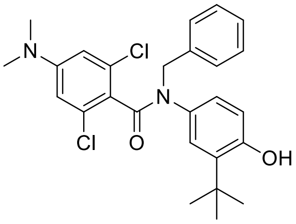	IC_{50} = 3.4 μM	AlphaScreen-based assay	(Xu et al., 2015)

Tabel S2. Tc values of the six newly identified hits to the 15 known FXR antagonists.

**Compd.**	**1a**	**2a**	**3a**	**3c**	**3d**	**3e**
**4**	0.32	0.20	0.11	0.13	0.11	0.11
**5**	0.30	0.19	0.10	0.09	0.08	0.10
**6**	0.26	0.15	0.11	0.13	0.09	0.09
**7**	0.28	0.17	0.11	0.13	0.09	0.09
**8**	0.38	0.20	0.13	0.10	0.08	0.11
**9**	0.27	0.19	0.15	0.10	0.08	0.11
**10**	0.15	0.16	0.21	0.23	0.27	0.22
**11**	0.11	0.17	0.31	0.30	0.30	0.24
**12**	0.08	0.25	0.23	0.20	0.13	0.17
**13**	0.13	0.21	0.23	0.20	0.19	0.15
**14**	0.18	0.25	0.26	0.22	0.27	0.19
**15**	0.09	0.14	0.17	0.15	0.12	0.12
**16**	0.18	0.20	0.20	0.20	0.23	0.24
**17**	0.31	0.21	0.15	0.17	0.18	0.13
**18**	0.15	0.19	0.26	0.26	0.21	0.31

References

Carter, B.A., Taylor, O.A., Prendergast, D.R., Zimmerman, T.L., Von Furstenberg, R., Moore, D.D., et al. (2007). Stigmasterol, a soy lipid-derived phytosterol, is an antagonist of the bile acid nuclear receptor FXR. *Pediatr. Res.* 62(3), 301-306. doi: 10.1203/PDR.0b013e3181256492.

Choi, H., Hwang, H., Chin, J., Kim, E., Lee, J., Nam, S.J., et al. (2011). Tuberatolides, potent FXR antagonists from the Korean marine tunicate botryllus tuberatus. *J. Nat. Prod.* 74(1), 90-94. doi: 10.1021/np100489u.

Di Leva, F.S., Festa, C., D'Amore, C., De Marino, S., Renga, B., D'Auria, M.V., et al. (2013). Binding Mechanism of the Farnesoid X Receptor Marine Antagonist Suvanine Reveals a Strategy To Forestall Drug Modulation on Nuclear Receptors. Design, Synthesis, and Biological Evaluation of Novel Ligands. *J. Med. Chem.* 56(11), 4701-4717. doi: 10.1021/jm400419e.

Dussault, I., Beard, R., Lin, M., Hollister, K., Chen, J., Xiao, J., et al. (2003). Identification of gene-selective modulators of the bile acid receptor FXR. *J. Biol. Chem.* 278(9), 7027.

Huang, H., Yu, Y., Gao, Z.T., Zhang, Y., Li, C.J., Xu, X., et al. (2012). Discovery and optimization of 1,3,4-trisubstituted-pyrazolone derivatives as novel, potent, and nonsteroidal farnesoid Xreceptor (FXR) selective antagonists. *J. Med. Chem.* 55(16), 7037-7053. doi: 10.1021/jm3002718.

Kainuma, M., Makishima, M., Hashimoto, Y., and Miyachi, H. (2007). Design, synthesis, and evaluation of non-steroidal farnesoid X receptor (FXR) antagonist. *Bioorgan. Med. Chem.* 15(7), 2587-2600.

Liu, P., Xu, X., Chen, L.L., Ma, L., Shen, X., and Hu, L.H. (2014). Discovery and SAR study of hydroxyacetophenone derivatives as potent, non-steroidal farnesoid X receptor (FXR) antagonists. *Bioorgan. Med. Chem.* 22(5), 1596-1607. doi: 10.1016/j.bmc.2014.01.032.

Nam, S.J., Ko, H., Shin, M., Ham, J., Chin, J., Kim, Y., et al. (2006). Farnesoid X-activated receptor antagonists from a marine sponge Spongia sp. *Bioorg. Med. Chem. Lett.* 16(20), 5398-5402. doi: 10.1016/j.bmcl.2006.07.079.

Nam, S.J., Ko, H.S., Ju, M.K., Hwang, H.S., Chin, J.W., Ham, J., et al. (2007). Scalarane sesterterpenes from a marine sponge of the genus spongia and their FXR antagonistic activity. *J. Nat. Prod.* 70(11), 1691-1695. doi: 10.1021/np070024k.

Song, K.B., Xu, X., Liu, P., Chen, L.L., Shen, X., Liu, J.H., et al. (2015). Discovery and SAR study of 3-(tert-butyl)-4-hydroxyphenyl benzoate and benzamide derivatives as novel farnesoid X receptor (FXR) antagonists. *Bioorgan. Med. Chem.* 23(19), 6427-6436. doi: 10.1016/j.bmc.2015.08.021.

Tsai, C.J., Liang, J.W., and Lin, H.R. (2012). Sesquiterpenoids from Atractylodes macrocephala act as farnesoid X receptor and progesterone receptor modulators. *Bioorg. Med. Chem. Lett.* 22(6), 2326-2329. doi: 10.1016/j.bmcl.2012.01.048.

Wu, J., Xia, C.S., Meier, J., Li, S.Z., Hu, X., and Lala, D.S. (2002). The hypolipidemic natural product guggulsterone acts as an antagonist of the bile acid receptor. *Mol. Endocrinol.* 16(7), 1590-1597. doi: DOI 10.1210/me.16.7.1590.

Xu, X., Lul, Y., Chen, L.L., Chen, J., Luo, X.M., and Shen, X. (2013). Identification of 15d-PGJ(2) as an antagonist of farnesoid X receptor: Molecular modeling with biological evaluation. *Steroids* 78(9), 813-822. doi: 10.1016/j.steroids.2013.04.018.

Xu, X., Xu, X., Liu, P., Zhu, Z.Y., Chen, J., Fu, H.A., et al. (2015). Structural basis for small molecule NDB (N-benzyl-N-(3-(tert-butyl)-4-hydroxyphenyl)-2,6-dichloro-4-(dimethylamino) benzamide) as a selective antagonist of farnesoid X receptor alpha (FXR alpha) in stabilizing the homodimerization of the receptor. *J. Biol. Chem.* 290(32), 19888-19899. doi: 10.1074/jbc.M114.630475.

Yu, D.D., Lin, W.W., Forman, B.M., and Chen, T.S. (2014). Identification of trisubstituted-pyrazol carboxamide analogs as novel and potent antagonists of farnesoid X receptor. *Bioorgan. Med. Chem.* 22(11), 2919-2938. doi: 10.1016/j.bmc.2014.04.014.
